# Computational Sentence‐Level Metrics of Reading Speed and Its Ramifications for Sentence Comprehension

**DOI:** 10.1111/cogs.70092

**Published:** 2025-07-22

**Authors:** Kun Sun, Rong Wang

**Affiliations:** ^1^ School of Foreign Languages Tongji University; ^2^ Department of Linguistics University of Tübingen; ^3^ NLP Institute University of Stuttgart

**Keywords:** Multilingual LLMs, Entire sentence processing, Sentence surprisal, Semantic relevance, Dual mechanism

## Abstract

The majority of research in computational psycholinguistics on sentence processing has focused on word‐by‐word incremental processing within sentences, rather than holistic sentence‐level representations. This study introduces two novel computational approaches for quantifying sentence‐level processing: sentence surprisal and sentence relevance. Using multilingual large language models (LLMs), we compute sentence surprisal through three methods, chain rule, next sentence prediction, and negative log‐likelihood, and apply a “memory‐aware” approach to calculate sentence‐level semantic relevance based on convolution operations. The sentence‐level metrics developed are tested and compared to validate whether they can predict the reading speed of sentences, and, further, we explore how sentence‐level metrics take effects on human processing and comprehending sentences as a whole across languages. The results show that sentence‐level metrics are highly capable of predicting sentence reading speed. Our results also indicate that these computational sentence‐level metrics are exceptionally effective at predicting and explaining the processing difficulties encountered by readers in processing sentences as a whole across a variety of languages. The proposed sentence‐level metrics offer significant interpretability and achieve high accuracy in predicting human sentence reading speed, as they capture unique aspects of comprehension difficulty beyond word‐level measures. These metrics serve as valuable computational tools for investigating human sentence processing and advancing our understanding of naturalistic reading. Their strong performance and generalization capabilities highlight their potential to drive progress at the intersection of LLMs and cognitive science.

## 1 Introduction

In the study of how humans comprehend and process language, computational models and metrics are essential for uncovering links between linguistic features and behavioral or neural responses. These tools enable linguistic prediction, modeling of language features, and specification of processing steps that can be quantitatively compared with behavioral and neural data.

One such model measures the information communicated by any particular linguistic component when considered within its left context. This method is commonly known as *surprisal*. *Word surprisal* estimates the information among words in context, and this metric has proven to be effective in predicting human word processing (Hale, 2001; Levy, 2008). In contrast to expectation‐based surprisal models, memory‐based models, such as those proposed by Lewis and Vasishth (2005) and Lewis, Vasishth, and Van Dyke (2006), focus on storing and retrieving information from prior input through underlying memory mechanisms, often grounded in frameworks like ACT‐R (Anderson, 1975). A representative metric in this category is *semantic similarity*, which estimates language processing difficulty by assessing the semantic relatedness of a word to its surrounding context (Hollis & Westbury, 2016; Mitchell & Lapata, 2010).

Word surprisal provides empirical support for the position that words are more difficult to process when they are harder to anticipate from preceding context (Demberg & Keller, 2008; Hale, 2016; Smith & Levy, 2013; Shain, Blank, van Schijndel, Schuler, & Fedorenko, 2020). However, recent work has shown that surprisal computed by neural language models tends to underpredict human reading time of both targeted constructions and naturalistic discourse (Arehalli, Dillon, & Linzen, 2022; Van Schijndel & Linzen, 2021). Recent work has also demonstrated the complexity of how neural language models compute surprisal and their relationship to human processing (Huang et al., 2024; Oh & Schuler, 2023). Although word surprisal remains effective for predicting word‐level sentence processing, there is an opportunity to extend these insights to holistic sentence‐level processing for further exploration. For example, next‐sentence prediction has been widely explored and shown to influence human language comprehension and processing (Rezaii et al., 2023;Yu, Gu, Huang, & Li, 2024; Zhou, Zhou, Long, Flinker, & Lu, 2025).

On the other hand, the memory‐based theory posits that processing difficulty arises from storing, retrieving, and integrating previous context with new input. For example, while the dependency‐locality model explains certain syntactic structures' processing (Gibson, 1998; Vasishth & Lewis, 2006), it has not been as effective as surprisal in predicting humans' contextual word processing (Boston, Hale, Kliegl, Patil, & Vasishth, 2008; Demberg & Keller, 2008; Levy, 2008). Another memory‐based metric, *semantic similarity*, gauges the similarity between the meanings of two words or phrases and is effective in predicting how words are processed. Further, *contextual semantic similarity*, which was developed from semantic similarity, concerns the semantic relatedness between a target word and its contextual words. Substantial empirical evidence supports the effects of word‐level semantic similarity in human language comprehension (Broderick, Anderson, Di Liberto, Crosse, & Lalor, 2018; Roland, Yun, Koenig, & Mauner, 2012; Sun, Wang, & Lu, 2023; Sun, Wang, & Baayen, 2025). However, recent research indicates that semantic relatedness at the discourse level also plays a significant role in human language comprehension and processing. (e.g., Carter & Hoffman, 2024; A. G. Lewis, Schoffelen, Hoffmann, Bastiaansen, & Schriefers, 2017).

Although the related studies have claimed that some given computational metrics are predictive of sentence processing by humans, the fact is that some of these metrics can only predict how words are processed in a given context (such as *word surprisal*) (De Varda & Marelli, 2023; Wilcox, Pimentel, Meister, Cotterell, & Levy, 2023; Hale, 2016). Focusing solely on word‐level sentence processing provides a granular perspective but may overlook emergent properties of sentence comprehension and processing. Although evidence does not suggest that humans process entire sentences at once, interactions between words (e.g., syntactic dependencies and semantic coherence) could introduce additional processing demands beyond what is captured by summing word‐level difficulties. Further research is required to explore the potential for over‐additive effects (Boston et al., 2008; Kuperberg, 2007; Warren & Gibson, 2002), where sentence‐level complexity exceeds the sum of its word‐by‐word components. In other words, we should also explore how humans comprehend and process entire sentences, rather than merely the individual words within them. In this context, sentence‐level metrics are potentially useful in understanding entire sentence processing by humans. For instance, the metric of contextual sentence‐level relevance computed by large language models (LLMs) could predict how Chinese natives comprehend sentences as a whole (Sun & Wang, 2023). In short, transitioning from word‐level to sentence‐level metrics allows for deeper insights into sentence processing, particularly in the context of discourse‐level comprehension.

During naturalistic discourse reading, sentence processing is influenced by various factors, including expectations about upcoming content based on preceding context and memory mechanisms that integrate new input through semantic relatedness. Surprisal and semantic relevance capture these dimensions: surprisal quantifies the information content and predictability of a linguistic unit, while semantic relevance reflects its degree of relatedness to surrounding contextual units. Sentence processing involves not only the incremental processing of words but also the resolution of syntactic structures, semantic coherence, and expectations about upcoming content: factors that may introduce over‐additive effects beyond the sum of word‐level difficulties (Boston et al., 2008; Warren & Gibson, 2002). Specifically, while word‐level surprisal effectively predicts processing difficulty for individual words based on their predictability in context, it may not fully capture the cognitive demands of comprehending sentences as integrated units within discourse. Instead, sentence‐level surprisal aims to quantify this holistic unpredictability. We expect that this metric could reveal processing demands unique to sentence‐level integration, complementing word‐level surprisal and advancing our understanding of naturalistic reading processes.

Sentence‐level semantic relevance is motivated by research in human sentence processing, which suggests that comprehension difficulty arises not only from unpredictability but also from the cognitive effort required to retrieve and integrate prior context with new linguistic input (Lewis & Vasishth, 2005; Lewis et al., 2006). We propose that a sentence's semantic relevance to its surrounding discourse, calculated as the sum of weighted similarities between the target sentence and its contextual sentences, reflects the cognitive ease of this integration process. High semantic relevance indicates strong coherence with preceding context, which can reduce memory demands and facilitate faster reading. In contrast, low relevance may increase processing difficulty due to weaker contextual support. Together, sentence surprisal and semantic relevance capture complementary aspects of comprehension: surprisal reflects probabilistic expectations, while relevance captures thematic continuity.

With recent advances in deep learning and the availability of large‐scale datasets, it has become increasingly feasible to develop and apply sentence‐level metrics. Using multilingual LLMs, we estimate the probability of a subsequent sentence (i.e., sentence surprisal) and compute semantic similarity between sentences (i.e., sentence relevance). To account for limitations in sentence length and human memory capacity, we consider a limited number of surrounding sentences (typically three to four) as contextual input when calculating these metrics. This approach is described as “memory‐aware” which operates similarly to the “attention” mechanism in Transformer architectures (Bahdanau, Cho, & Bengio, 2014; Vaswani et al., 2017) by selectively incorporating relevant contextual information. The present study aims to compute these sentence‐level metrics, test hypotheses regarding their cognitive basis, and investigate their potential influence on human sentence processing.

We aim to apply these computational metrics to evaluate their predictive power across multiple languages, extending beyond English. Fortunately, both suitable datasets and computational tools are available. For example, existing multilingual LLMs are well‐equipped to process and understand texts in a variety of languages, making them effective for computing the proposed sentence‐level metrics. Additionally, multilingual resources on language comprehension, such as the Multilingual Eye‐movement Corpus (MECO), offer valuable empirical data. The MECO comprises eye‐tracking data collected from participants reading texts in 13 different languages (Siegelman et al., 2022), making it an ideal testing ground for our study. The current study first computes sentence‐level metrics and examines their influence on sentence reading speed. By employing advanced statistical methods, we expect these metrics to yield accurate predictions of sentence‐level processing and comprehension. To further assess the robustness and generalizability of our approach, we will conduct a cross‐linguistic investigation.

## 2 Related work

*Word surprisal*, defined as the negative logarithm of a word's probability given its left context within a sentence, surprisal=−logp(word∣left context), has been widely used to predict word‐level processing difficulty (Hale, 2016). This metric signifies that predictable words require less cognitive effort to process. However, it remains an open question: which context aspects—lexical, syntactic, semantic, or conceptual—facilitate prediction. While word surprisal accounts for the influence of the left context, this context is confined to a single sentence. Word surprisal has primarily been used to predict individual word processing times, such as the duration of reading a word. However, it falls short in predicting sentence‐level measures, such as overall reading speed. Therefore, our goal is to develop metrics that go beyond “within‐sentence” analysis to predict reading speed. Further, *surprisal* is defined as the negative logarithm of the probability of an event, and it can be applied across various linguistic levels, including phonemes, words, sentences, and discourse (Gwilliams & Davis, 2022; Pimentel et al., 2021; Venhuizen, Crocker, & Brouwer, 2019). This theoretical flexibility supports the validity of sentence‐level surprisal. Additionally, in linguistics, surprisal is a measure of how predictable a linguistic unit is within a given context. It is a broader concept than simply predicting the next linguistic unit using a left‐to‐right window. Although surprisal is often discussed in the context of next‐unit prediction, this is not its only application. Bidirectional language models like BERT can also be used to compute surprisal in a general sense. This allows for a flexible and comprehensive estimation of predictability that goes beyond traditional left‐to‐right approaches.

Moreover, the rapid advancement of language models has transformed the methods used to calculate word probabilities, shifting from traditional n‐gram models to Transformer‐based architectures (Goodkind & Bicknell, 2018; Schrimpf et al., 2021; Wolf et al., 2020; G. Wilcox, Gauthier, Hu, Qian, & Levy, 2020). These Transformer‐based models provide the computational capacity to estimate sentence‐level metrics with high accuracy. Employing LLMs, we can compute sentence‐level surprisal across multiple languages with both theoretical soundness and empirical precision. Additionally, Rosen and Dale (2024) adopted a comparable LLM‐based approach, using an informal mathematical argument to connect it to an empirical study that links semantic meaning with global lexical prediction. This supports the idea that our sentence‐level surpsial, particularly those based on the chain rule, implicitly capture token‐level information.

On the other hand, semantic similarity or relevance at the word level has been shown to predict human processing ease by reflecting how well a word fits its context (Roland et al., 2012; Sun et al., 2023). Extending this to the sentence level, we hypothesize that semantic relevance among sentences similarly eases processing or comprehension by supporting discourse coherence. Our “memory‐aware” approach operationalizes this by weighting the similarities of contextual sentences based on their proximity. Some effective methods have been proposed for computing semantic relevance among words within a sentence. These approaches involve integrating contextual information using the “attention‐aware” method, resulting in more powerful contextual semantic relevance (Sun et al., 2023; Sun et al., 2025). Nevertheless, we believe that the similar methods can be extended and applied to process entire sentences rather than merely words. By modifying the “attention‐aware” approach, we can calculate the semantic relevance among sentences, enabling us to measure the extent to which a target sentence is semantically connected to its neighboring sentences within discourse.

The present study investigates how sentence‐level metrics take effect on reading speed. Reading speed, typically measured in words per minute (i.e., wpm), reflects the rate at which a person reads text or sentences and serves as an indicator of reading fluency and efficiency. Influenced by reader skill, text complexity, text coherence, and purpose, reading speed has been widely studied (Carver, 1990; Jackson & McClelland, 1979; Herman, 1985; Rayner, Slattery, & Bélanger, 2010). Further, reading speed and sentence/text comprehension are closely interconnected, shaped by various cognitive factors. Research shows a positive correlation between reading speed and comprehension levels, where higher reading speeds are often associated with better comprehension (Blommers & Lindquist, 1944; Breznitz, 2006; Miyata, Minagawa‐Kawai, Watanabe, Sasaki, & Ueda, 2012; Pertiwi & Sujarwati, 2023; Rasinski, 2000). However, this relationship is non‐linear. For instance, reading too slowly can impede comprehension due to memory constraints, while overly rapid reading may not allow adequate processing time (GEMR, 2023). Additionally, strong syntactic awareness facilitates the parsing of complex sentence structures, improving overall understanding. Semantic context also accelerates sentence reading, demonstrating the influence of top‐down cognitive processes (Primativo, Spinelli, Zoccolotti, De Luca, & Martelli, 2016). Nonetheless, sentence‐level metrics have seldom been taken to explore their effects on reading speed. We believe that our proposed sentence‐level metrics could predict human sentence reading speed. By examining their effects on reading speeds, we can gain insights into how sentence‐level metrics influence text comprehension.

In addition, reading difficulties also stem from sources such as word‐level factors, syntactic structure, and sentence‐level elements. While context significantly affects sentence processing, effective computational metrics are needed to evaluate this. Sentence comprehension and processing involve the mental processes when understanding language utterances, significantly influenced by context and other factors. Although studies have examined various aspects of comprehension such as word processing and syntactic integration (Altmann & Mirković, 2009; Carpenter, Miyake, & Just, 1995; Gibson, 1998; Hale, 2016; Kamide, 2008; McRae & Matsuki, 2013; Rohde, 2002), there is a dearth of computational methods to measure context impact and research on comprehending sentences as a whole. The current study aims to address this gap by developing computational sentence‐level metrics for evaluating entire sentences across languages.

All things considered, entire sentence processing may be influenced by factors like sentence expectations from previous context and memory integration based on semantic relatedness. Word‐level surprisal and semantic relevance have been shown to independently affect the processing of words in context. The current study explores whether sentence‐level surprisal and semantic relevance are useful in processing sentences as a whole. Additionally, research on computational approaches to cognitive science has predominantly focused on the English language, leading to a lack of investigation in other languages to test the generalizability (Blasi, Henrich, Adamou, Kemmerer, & Majid, 2022). It is essential to enable cross‐lingual generalizations using computational models or metrics in the present study.

## 3 Material and method

### 3.1 Testing datasets

Multilingual databases enable cost‐effective and reliable assessment of cognitive and neural aspects of language processing. Among them, the MECO is particularly valuable, as it provides eye‐tracking data across 13 diverse languages, offering insights into cognitive effort during sentence processing (Siegelman et al., 2022). Each language's participants read 12 encyclopedia entries of 2000 tokens, resulting in 36,000 tokens in total. These entries were similarly complex across languages and generated about 70,000 to 80,000 eye‐tracking data points per language. The MECO was chosen for its language diversity, large native speaker counts, and ample eye‐tracking data. *Reading speed (or rate)* has been extensively explored, typically employing text or individual sentences as the unit of interest (Biancarosa, 2005; Brysbaert, 2019; Carver, 1976; Miller & Coleman, 1971; Siegelman et al., 2022).

The present study focuses on *sentence reading speed* (= *the number of words in this sentence/total fixation duration of a sentence*) − *word number per second (for a sentence or text)*. “Sentence reading speed” can be calculated from the MECO dataset for various languages. When the reading speed is low, readers may use more time to process this sentence. In contrast, a higher sentence reading speed indicates that less time is used to process every word in this sentence. While reading speed is not a direct oculomotor measure, it is influenced by oculomotor factors such as fixation duration, saccade durations, and the tendency to skip words. The reading speed is also interconnected with these oculomotor measures, as they collectively contribute to how quickly and efficiently one can read and understand a sentence or text. Fixation duration for a word merely represents the difficulty of an individual word. In contrast, the reading speed is considered as the overall difficulty when readers comprehend a sentence/text as a whole.

While reading speed measures the number of words processed per minute or second across sentences, word reading duration assesses the processing difficulty of a single word. As these measures are fundamentally different, word surprisal predicts the processing of individual words and does not extend to sentence‐level processing (Additional details are provided in Section A of the Supporting Information (SI), with an indication included in the printed version of the main text that the SI is available.). It is essential to develop practical sentence‐level metrics to evaluate their potential impact on reading speed. The following sections detail the computation methods for these metrics.

### 3.2 Computing sentence surprisal

We employed two multilingual LLMs: m‐BERT and mGPT to compute sentence surprisal. Sentence surprisal is the negative logarithm of next sentence probability, −log(p(sentence∣left context)), which is similar to word surprisal. In theory, Multi‐lingual BERT (m‐BERT) (Devlin, Chang, Lee, & Toutanova, 2018) can be employed to compute word‐level or sentence‐level surprisal for various languages. BERT, a masked language model, has good performance in a number of Natural Language Processing (NLP) tasks (Kalyan, Rajasekharan, & Sangeetha, 2021; Salazar, Liang, Nguyen, & Kirchhoff, 2019) and can be used to estimate next word probabilities. We used the state‐of‐the‐art m‐BERT model (i.e., multilingual‐bert‐uncased) because it can be consistently applied in different languages in order to compute *word/token probability*. However, directly computing sentence probability with m‐BERT is challenging without using its “next sentence prediction (NSP)” mechanism. Fortunately, we can take some strategies to allow m‐BERT to approximate next sentence probabilities. For instance, we gauged the joint probability of an entire sentence conditioned on its preceding context. This is achieved by using the chain rule of probability, breaking the sentence into individual tokens, and computing the probability of each token given all preceding tokens (and the context).

Further, GPT is essentially an autoregressive model based on the Transformer architecture, trained on a language modeling task, where the objective is to predict the next word in a sequence given the preceding words. The model learns to assign probabilities to words based on the context. The method of the chain rule can be introduced in applying mGPT to compute sentence surprisal (Shliazhko et al., 2022). The difference is that GPT employs the language modeling to compute word probability, but BERT uses a masked language model to calculate word probability.

To compute sentence surprisal, we proposed three methods to estimate the metric. The three methods are summarized as follows: the first one is to use the *chain rule (CR)*, which was mentioned above. Sentence surprisal is calculated by tokenizing a sentence into individual elements and computing the joint probability of the sentence given a context. This is done by multiplying the conditional probabilities of each token given its preceding tokens and the context, applying the CR of probability to language sequences. The method can be computed based on either m‐BERT or mGPT. The second method is to use the *NSP* mechanism in m‐BERT to compute sentence surprisal. Third, sentence surprisal is also quantified by computing the *negative log‐likelihood (NLL)* of the sentence when conditioned on its context. NLL is commonly used as a loss function to evaluate how well a model's predicted probability distribution aligns with the actual observed data. For a given sequence of tokens, a higher NLL indicates greater model surprise, reflecting lower predicted probabilities for the true sequence. The details on executing the three methods are presented in Sections B and C of the SI.

Since the computation of sentence surprisal often involves elements of word surprisal, it is important to clarify the distinction between the two. We provide a brief comparison of word‐level and sentence‐level surprisal to elucidate their differences in processing difficulty, computational methods, and roles as predictors in statistical modeling. Word surprisal quantifies the unpredictability of individual words within a sentence, capturing local processing difficulty. In contrast, sentence surprisal is computed as the joint probability of an entire sentence given its preceding context. This metric better reflects global processing difficulty by incorporating sentence‐level features such as syntactic complexity and discourse expectations, which word‐level metrics may overlook. From a computational perspective, word surprisal is derived from probabilities confined to a single sentence, whereas sentence surprisal integrates probabilities over a broader context. In statistical models, these two types of surprisal correspond to different response variables: word surprisal is often associated with fixation durations on specific words, while sentence surprisal aligns more closely with full‐sentence reading times. Moreover, the control variables used in models of word and sentence surprisal differ, highlighting their distinct explanatory power. Further details on the differences between sentence‐level and word‐level surprisal can be found in Section B of the SI.

**Fig. 1:**
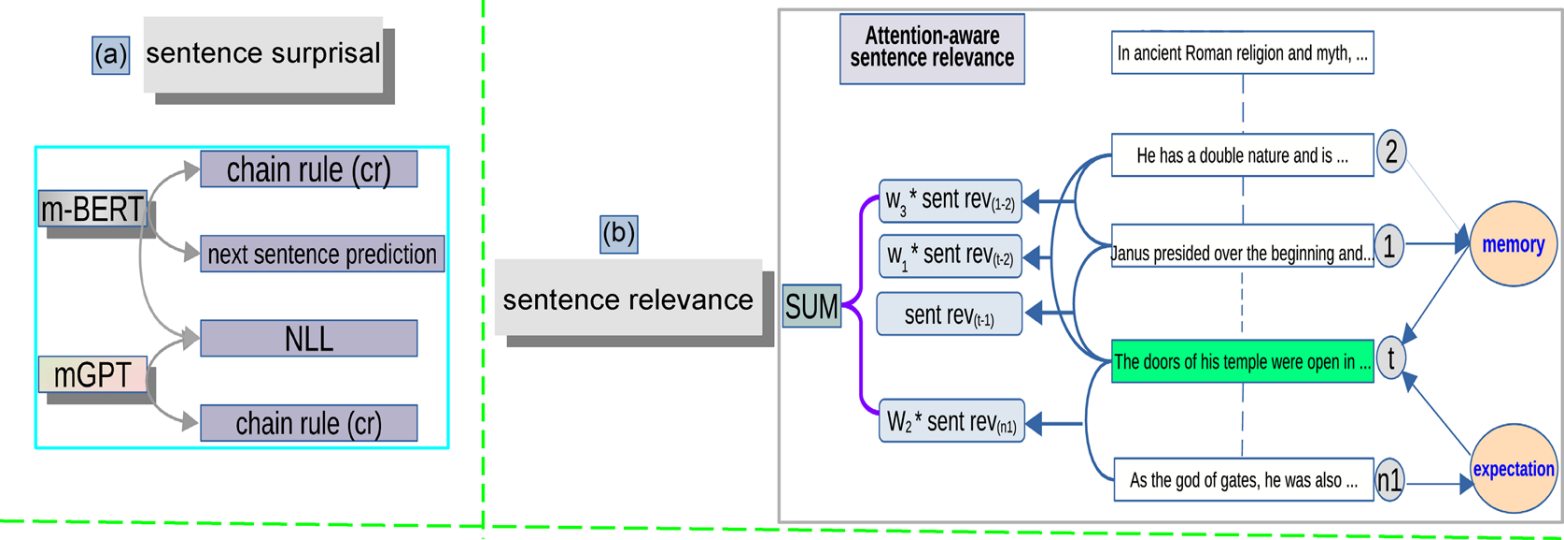
The computational methods in the present study. *Note*: Panel A illustrates sentence surprisal, while panel B depicts sentence relevance.

In general, we employed three methods for computing sentence surprisal, based on two multilingual LLMs: m‐BERT and mGPT. “Sentence surprisal” quantifies *the level of unpredictability or information associated with encountering a given sentence*. The CR and the NLL approaches were applied to the two LLMs for this purpose. In contrast, the NSP method was exclusively implemented on m‐BERT. The methods and models are shown in panel A of Fig. 1.

### 3.3 Computing memory‐aware sentence relevance

This section elaborates on the computation of “sentence semantic relevance” (shortened as “sentence relevance”). Consider a four‐sentence window, as shown in panel B of Fig. 1, where the goal is to evaluate the semantic relationship between the target sentence and its surrounding context, which includes the two preceding sentences and the one following sentence within this sliding window. The computation unfolds in two primary steps. Initially, embeddings for each sentence within the window are generated using either m‐BERT or mGPT. Subsequently, the application of cosine similarity to the sentence embeddings derived from m‐BERT or mGPT serves as a conventional approach to ascertain the semantic similarity between any pair of sentences^1^, as detailed in Section D of the SI. Nonetheless, this step is to obtain the relevance of two sentences in this window. Our objective, however, is to compute the semantic relatedness between a target sentence and its contextual sentences, which is another sentence‐level metric.

After computing the semantic similarity values for each pair of sentences within the window, we applied a “memory‐aware” approach to process the four resulting similarity scores. This approach is designed to address the cognitive memory bottleneck and account for memory constraints, as discussed in Futrell, Gibson, and Levy (2020), thereby grounding the method in memory‐based processing principles. In practice, the “memory‐aware” approach is implemented as a convolution‐based, *recency‐weighted* algorithm. It uses convolution as a core operation, a technique commonly applied in signal processing, computer vision, and engineering, to emphasize more recent information while still incorporating earlier context for local feature extraction (Burrus & Parks, 1991; Li, Liu, Yang, Peng, & Zhou, 2021). Convolution is crucial for identifying and emphasizing local patterns in data. In the context of cognition, the convolution‐based method could model the gradual integration of contextual information, capture processing spillover effects, and reflect cognitive load arising from recently encountered difficult words. This method has been successfully applied to compute word‐level metrics for predicting human reading behavior (Sun et al., 2023; Sun & Liu, 2025; Sun et al., 2025). The following provides the technical details of the computation.

The sliding window comprises four sentences (2, 1, *t*, and *n1*), as illustrated in panel B of Fig. 1. Here, *t* denotes the target sentence and *n1* indicates the subsequent sentence. Sentence 1 is the immediately preceding sentence, and 2 refers to the sentence prior to that. Our goal is to evaluate the semantic relationship between the target sentence (*t*) and the surrounding context (sentences 2, 1, and *n1*). Specifically, the “sentence relevance” (sentrev) is computed as the weighted sum of cosine similarities (sim) between the target sentence t and its contextual sentences $c_{i}$, where i∈{2,1,t,n1} corresponds to the target sentence itself, the immediate preceding sentence, the second preceding sentence, and the following sentence, respectively. The weights are w=[1,0.5,0.3]. It is formalized as (1):

$$\mathrm{sentrev}=\sum_{i\in \{2,1,t,n1\}}w_{i}\cdot \text{sim}\left(t,c_{i}\right),$$

with weights w=[1,0.5,0.3] for $c_{2},c_{1},c_{n1}$. This formulation emphasizes the influence of contextual sentences based on their positional distance, reflecting cognitive recency effects. We assume that the semantic contributions of the three contextual units should ideally be balanced (each contributing one‐third). However, the sentence immediately adjacent to the target sentence is assigned a disproportionately larger weight (e.g., 1), reflecting a non‐linear decline in semantic relevance with increasing distance. Sentences that are farther away, such as the second preceding sentence, are given smaller weights (e.g., 0.7). This exponential weighting highlights the stronger influence of nearby context compared to more distant linguistic units. Note that the self‐similarity of the target sentence ($c_{t}$) is not included in Equation (1).

**Fig. 2:**
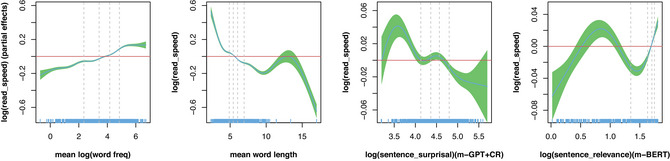
The overall partial effects of the primary predictors (sentence surprisal and sentence relevance) on reading speed across languages. The *x*‐axis denotes the metric, and the *y*‐axis represents the reading speed. Reading speed, sentence surprisal, and sentence relevance are transformed by logarithm (marked as “log_”) in order to get closer normal distribution, further having better fittings. Note that sentence surprisal and sentence relevance were not modeled with the “by = language” term in the GAMM. Each curve in one plot depicts the relationship between a predictor variable and the response variable of reading speed. Steeper slopes on these curves indicate a stronger relationship between the predictor and reading speed, while flatter slopes suggest a weaker effect.

The “memory‐aware” approach weights the semantic similarity between a target sentence and its contextual sentences (e.g., the preceding two and following one) based on their positional distance, with closer sentences receiving higher weights (e.g., 0.5 for immediate neighbors, 0.3 for non‐neighbors). This weighting scheme is inspired by cognitive evidence that recent context exerts a stronger influence on sentence comprehension due to working memory constraints and recency effects (Gibson, 1998; Lewis et al., 2006). The decaying weights in our memory‐aware approach are similar to the human forgetting curve (Loftus, 1985), shown in Fig. 2 (the execution strategies and the motivations of using “memory‐aware” from cognitive and mathematical perspectives are provided in Section D of the SI). Unlike the attention mechanism in Transformers (Bahdanau et al., 2014; Vaswani et al., 2017), which learns weights via neural networks, our method uses fixed, interpretable weights to mimic how readers prioritize immediately preceding context over more distant information during naturalistic reading. This approach reflects the decay of contextual influence over a short discourse window, consistent with memory‐based processing dynamics. Each similarity value is multiplied by its corresponding weight, and the results are then aggregated to produce a single value. This value signifies *the semantic relevance of the target sentence within its context* (i.e., “sentence relevance”). Employing various sentence embeddings created by m‐BERT and mGPT, we are able to derive two distinct values of “sentence relevance” for the identical target sentence within its specific context. The computation is illustrated in panel B of Fig. 1.

The method proposed for computing “memory‐aware” metrics was not involved in introducing attention layers in the Transformer. The term “memory‐aware” sounds similar to attention because the method works as well as attention in incorporating contextual information. The “memory‐aware” approach can work as a memory agent (see Section D of the SI). This approach considers both preceding and following sentences as potential sources of contextual information. The relationship between these contextual sentences and the target sentence is then weighted based on their positional distance and aggregated to create a comprehensive metric of semantic relevance within the discourse. This “memory‐aware” approach is highly explainable from linguistic, cognitive, and mathematical perspectives. (More details are provided in Section D of the SI.) Table 1 provides an overview of the metrics in our analysis.

**Table 1:** Methods, metrics, and statistical analysis

Method	Metric	Computation	Statistical analysis
Sentence surprisal	‐ Chain rule ‐ Next sentence prediction ‐ Negative Log‐Likelihood	$-\log\left(p(\text{sentence}|\text{left context})\right)$	Generalized additive mixed models
Memory‐aware relevance	Sentence‐level semantic relevance	$\sum {\text{Sim}}_{\left(t,c\right)}\cdot W_{\left(t,c\right)}$ (t: target sentence, c: contextual sentence, W: weights)	

### 3.4 Statistical method and model comparison

We employed generalized additive mixed models (GAMMs) (Wood, 2017) to investigate the predictive power of these metrics on sentence reading speed. GAMMs extend linear mixed‐effects models by capturing non‐linear relationships using smooth functions, making them ideal for modeling complex patterns while still accounting for random effects. The present study allows GAMM fittings to include these control predictors (“mean word length,” “mean word frequency” for a sentence^2^) and random variables (e.g., languages, participants). Including these variables is crucial for achieving optimal GAMM fittings.

The Akaike information criterion (AIC) is used in GAMMs to compare model fit while penalizing complexity. Lower AIC values indicate a better balance between goodness‐of‐fit and model simplicity, helping to identify the most parsimonious model among competing alternatives. The performance of GAMM models was evaluated using the difference in AIC, denoted as Δ AIC, between the base and full GAMM. This metric serves as a measure of model effectiveness, calculated as Δ AIC = AIC of full model − AIC of base model. A smaller (negative) Δ AIC value indicates that the metric of our interest provides more accurate predictions of fixation duration compared to the baseline model. More details on statistical methods can be found in Section E of the SI.

## 4 Results

### 4.1 Overall performance

We fitted eight GAMM models, each using one of the sentence‐level metrics as the main predictor of the response variable, “sentence reading speed”. The GAMM models also include “mean word length” and “mean word frequency” as the control predictors and “participant” as a random effect. This is what an optimal GAMM formula looks like

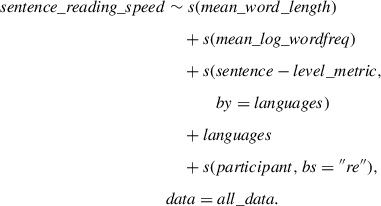




With “by = languages” in the GAMM model, it facilitates the inclusion of responses at each language level in the model as distinct terms. Concurrently, smooths by “languages” are employed to capture and model the variations around these language‐specific responses; re = random effect, random slope adjusting the slope of the trend of a numeric predictor.

The base GAMM model (without metrics of our interest) is depicted as

$$\begin{array}{cc} \mathrm{sentence}\_\mathrm{reading}\_\mathrm{speed} & ∼s(\mathrm{mean}\_\mathrm{word}\_\mathrm{length})\\ & \;+s(\mathrm{mean}\_\log\_\mathrm{wordfreq})\\ & \;+\mathrm{languages}\\ & \;+s(\mathrm{participant},\mathrm{bs}=\text{”re”}). \end{array}$$

Note that the base model does not include the metric of interest. The variable “languages” is treated as a parametric fixed effect, in contrast to the smooth terms (e.g., s(mean_word_length)). As a categorical predictor, “languages” represents the language in which each sentence is written (e.g., Dutch, English, Spanish). Including this variable allows the GAMM to account for baseline differences in reading speed across languages, while also modeling smooth effects such as word length and word frequency, along with random effects like participant variability. This structure makes it possible to examine whether other predictors, such as surprisal or semantic relevance in the full model, explain additional variance in reading speed beyond what is attributable to language differences.

The ΔAIC represents the difference in AIC values between the full model and the base model, calculated as the AIC of the full model minus the AIC of the base model. Following the GAMM fittings, we present and interpret the results as follows.

First, we examined whether a variable is significant or not. We adopt the criterion that a variable is considered significant in a GAMM fitting if its *p*‐value is below .01. The results show that the control predictors, namely *mean word length* and *mean word frequency*, are consistently significant across all models. This indicates that the processing of sentences in the context of naturalistic discourse reading is significantly shaped by both the average length and frequency of the words employed. Specifically, the *mean word length* exerts a negative influence on the speed of sentence reading. In essence, sentences composed of longer words are read more slowly. Conversely, the *mean word frequency* positively impacts reading speed, meaning that sentences containing words that are more frequently used facilitate faster reading. Therefore, when the average frequency of words in a sentence is high, it enables readers to process the text more swiftly. The effects of word length and frequency are particularly notable in word processing during naturalistic discourse reading. As such, holistic sentence processing and word processing share a great deal of similarity. Moreover, each of the sentence‐level metrics (F > 30, Effective DF = 8.83) makes a very meaningful, though secondary, contribution to explaining variance in reading speed, with an effect approximately one‐third as strong as that of the primary predictors, lexical frequency and sentence length (F > 100).

The results of GAMM fittings also show that the majority of the metrics computed by either m‐BERT or mGPT are capable of predicting the reading speed quite well. The random effect of the *participant* is significant in all GAMM fittings. We further compared the performance of the GAMM fittings with different metrics. Table 2 presents the results on Δ AIC for comparing these GAMM fittings. A lower Δ AIC value indicates a better GAMM fitting. It also suggests that the computational metric performs better than others. The basis for comparison is the consistent data point numbers (*n* = 70,442) and identical elements in each GAMM fitting. As illustrated in Table 2, the result reveals that sentence surprisal, as calculated using three distinct methodologies, namely, CR, NLL, and NSP, proves to be effective in predicting reading speed. Among the evaluated metrics of sentence surprisal, the performance of mGPT, when applying the chain rule, stands out as the most effective. The computation of sentence relevance, whether through m‐BERT or mGPT, demonstrates viability in prediction accuracy. In summary, sentence relevance (computed using mBERT) outperformed all sentence surprisal metrics in predicting reading speed. Nevertheless, the inclusion of both sentence surprisal and sentence relevance as a smooth function into the GAMM fitting (the last row in Table 2) significantly enhances predictive performance, surpassing the results achieved when these metrics are applied independently.

**Table 2:** Comparison of GAMM fittings with different computational measures on MECO (*n* = 70,442)

GAMM Fittings (Sentence Reading Speed)	ΔAIC
Sentence surprisal (m‐BERT‐CR)	−1,389.83
Sentence surprisal (m‐BERT‐NSP)	−429.42
Sentence surprisal (m‐BERT‐NLL)	−1,690.61
Sentence surprisal (mGPT‐CR)	−1,718.3
Sentence surprisal (mGPT‐NLL)	−1,371.1
Sentence relevance (m‐BERT)	−1,794.5
Sentence relevance (mGPT)	−1,433.3
Sentence surprisal (m‐BERT‐CR) + Sentence relevance (m‐BERT)	−3,818.12

*Note*. A model with a smaller Δ AIC indicates better performance. CR, chain rule; NLL, negative log‐likelihood; NSP, next sentence prediction.

As shown in Table 2, the three methods (i.e., CR, NLL, and NSP) employing BERT can compute three types of sentence surprisal, which in turn can predict reading rates. While the CR method can also compute sentence surprisal and predict reading speed, its performance depends on which language model is based on. This suggests that BERT could also precisely model the human reading process when comprehending entire sentences, compared to GPT (more details are given in Section F of the SI). Furthermore, although the NSP method employing BERT operates within a two‐sentence window, it still manages to predict reading rates to a certain degree. However, the comparison between NSP and CR/NLL performance (Table 2) suggests that the human reading window during text processing may extend beyond two sentences. Additionally, sentence relevance computed by BERT‐generated embeddings outperforms all sentence surprisal metrics as well as that based on embeddings from GPT.

We selected the best‐performing metrics to visualize their overall partial effects. Fig. 2 presents the partial effects of the selected metrics, excluding the term *by = languages*, based on the GAMM fittings. Fig. 2 illustrates the partial effects of sentence surprisal and semantic coherence on reading speed in the right two plots, while the left two plots depict the partial effects of mean word frequency and mean word length, respectively. Each plot conveys several key details. The estimated partial effect of the predictor appears as a blue line, accompanied by the 99% confidence interval of the smooth, shaded in green. The approximate significance of the smooth is displayed in the upper center of each plot. Partial‐effect smooths are centered around a *abline*, and for any interval of predictor values where the confidence interval fluctuates around this line, the predictor has no significant effect. Vertical gray lines mark the quartiles of the predictor's distribution, and rugs along the *x*‐axis provide additional details on this distribution, particularly useful for identifying outliers. More information about how to read the partial effect is provided in the caption of Fig. 2.

As depicted in the left two plots of Fig. 2, “mean word frequency” exhibits a positive relationship with reading speed, whereas “mean word length” shows a negative relationship. Unlike reading duration, where longer durations indicate slower processing, higher reading speed reflects faster processing, leading to different trends for these metrics compared to those observed for reading duration. The right two plots of Fig. 2 reveal that sentence surprisal, estimated by m‐GPT+CR, generally displays a negative relationship with reading speed across most data points. This pattern is basically consistent with the known effect of word surprisal on word reading duration and aligns with expectations. Specifically, as sentence surprisal increases, it tends to reduce reading speed across the 13 languages examined, suggesting that higher surprisal is associated with slower reading. Conversely, sentence relevance demonstrates a positive association with reading speed, indicating that greater semantic relevance facilitates faster reading across these languages. This is consistent with the effect of word‐level semantic relevance (Sun et al., 2023; Sun & Liu, 2025).

### 4.2 Performance in individual languages

First, sentence surprisal and sentence relevance are fundamentally different metrics. Their overall correlation is low, with ρ=−0.054 (*p* < .001), and the correlation within each language is also consistently small. This confirms that the two metrics capture distinct aspects of sentence processing. Additional details can be found in Section G of the SI. Because of their low correlation, both metrics can be included in the same GAMM fitting without causing collinearity problems, as shown in the bottom row of Table 2. In contrast, some related studies could have encountered the potential collinearity issues by including multiple closely related surprisal‐based metrics, such as combining GPT‐based surprisal with n‐gram surprisal in a single GAMM model. Since these metrics are typically highly correlated, their simultaneous inclusion in a GAMM fitting increases the risk of collinearity (see Section E of the SI).

The results from fitting the GAMMs offer valuable insights into the importance of the variables considered. This section focuses on the performance of the selected outperformed metrics in each language (among 13 languages) using a similar GAMM analysis. The base model includes both metrics of our interest, and the data are only for one specific language. The optimal base GAMM model for each language is specified as follows:

$$\begin{array}{cc} \mathrm{reading}\_\mathrm{speed} & ∼s(\mathrm{mean}\_\mathrm{word}\_\mathrm{length})\\ & \;+s(\mathrm{mean}\_\log\_\mathrm{wordfreq})\\ & \;+s(\mathrm{participant},\mathrm{bs}=``re^{\prime \prime }),\\ & \;\text{data}=\mathrm{specific}\_\mathrm{language}\_\mathrm{data}. \end{array}$$

The Δ AIC is computed by comparing the base model (excluding both predictors) with models that include one predictor of interest. Specifically, for “sentence surprisal,” we subtract the AIC of the model including sentence surprisal from the AIC of the base model. Similarly, for “sentence relevance,” we subtract the AIC of the model including sentence relevance from the AIC of the base model. In this context, a smaller Δ AIC indicates that the predictor contributes more to model fit and thus exhibits stronger performance. The significance of either “sentence surprisal” or “sentence relevance” is assessed using the *p*‐value (the significance threshold being *p*‐value < .01), F value, and the shape of the partial‐effect curve. These metrics were selected to represent, respectively, the concepts of sentence surprisal and sentence relevance in our examination of each language. The results are illustrated in Fig. S3 in Section F of the SI. It turns out that sentence surprisal and sentence relevance show significance across 13 languages. The language‐specific trends of the two sentence‐level metrics, sentence surprisal and sentence relevance, align closely with their overall trends across the 13 languages. Table S1 in Section F of the SI compares the performance of the two sentence‐level metrics, sentence surprisal and sentence relevance, across each language. The results indicate that both metrics make comparable contributions to predicting reading speed across the 13 languages, with their language‐specific performance largely consistent with their overall trends in predicting human reading speed.

## 5 Discussion

### 5.1 Main findings

As highlighted in the Introduction and Related Work sections, word surprisal has been shown to reliably predict language comprehension across various datasets (Ryskin & Nieuwland, 2023). Although sentence prediction has been shown to influence language comprehension and processing (e.g., Goldstein et al., 2022; Kriegeskorte, Mur, & Bandettini, 2008), sentence‐level metrics have rarely been used as predictors in studies of human language comprehension and processing. In the present study, our main contribution is proposing practical, effective algorithms for computing sentence‐level metrics, namely sentence surprisal and sentence relevance. We evaluated their effectiveness using statistical modeling on a large‐scale, cross‐language eye‐movement dataset. The results of this study highlight the predictive strength of sentence‐level metrics, especially sentence surprisal and semantic relevance, in shaping sentence reading speed.

As shown in our results, sentence surprisal can serve as an effective predictor of the sentence reading speed. Specifically, if a sentence is highly predictable given its left context (i.e., lower sentence surprisal), readers have a higher/faster reading speed. It may indicate that sentences become easier to process. On the other hand, if a sentence is more unpredictable (i.e., higher sentence surprisal), this can lead to slower/lower reading speed and potentially disrupt the flow of reading. These findings suggest that sentence surprisal significantly influences reading speed across languages. The ability to predict upcoming sentences may be a fundamental aspect of language processing, enabling readers to anticipate and smoothly integrate new information with what has come before.

Numerous studies have also identified the influence of context on sentence processing (Cohen & Servan‐Schreiber, 1992; Grisoni, Miller, & Pulvermüller, 2017). However, the current study is the first to propose a method for computing sentence‐level semantic relevance. The sentence relevance based on the “memory‐aware” approach proves effective in interpreting and predicting reading speed, and it may also help anticipate the processing difficulties readers encounter when comprehending full sentences. When a sentence is more semantically relevant to the context in which it appears, it is more likely to be processed quickly and accurately and readers are more likely to understand its meaning without having to invest a lot of cognitive effort (i.e., higher reading speed). Conversely, when a sentence is irrelevant to the context (i.e., lower sentence relevance), it may slow down reading speed and require more cognitive effort. Overall, our findings demonstrate that sentence relevance has a profound effect on sentence reading speed. The following section explores how our metrics take effect on sentence comprehension in the context of our findings.

Additionally, as shown in Table 2, our analysis shows that sentence surprisal computed using CR outperforms the NLL method in predicting reading speed suggesting that CR aligns more closely with human incremental processing. This suggests that the CR method, which estimates sentence probability by sequentially predicting the next word and conditioning on the rest, may closely approximate the incremental nature of human sentence processing. For mBERT, however, the NLL method slightly outperforms CR, indicating that both approaches can be effective depending on the model architecture. Overall, these results highlight that while both CR and NLL capture aspects of sentence predictability, the CR tends to better reflect the cognitive dynamics involved in natural reading, especially in autoregressive‐style models like mGPT. Despite this, among all individual metrics, sentence relevance achieves the best performance, highlighting the importance of memory‐based semantic alignment in sentence processing.

### 5.2 Reading speed and sentence comprehension

Reading speed and sentence comprehension are intricately linked, shaped by a variety of complex cognitive factors (Blommers & Lindquist, 1944; Breznitz, 2006; Miyata et al., 2012; Pertiwi & Sujarwati, 2023; Rasinski, 2000). In the present study, empirical evidence from 13 languages supports the argument that our metrics significantly affect reading speed, providing insights into sentence comprehension.

Sentence reading speed could serve as an important indicator of sentence comprehension, reflecting the efficiency of cognitive processing. Our findings reveal that sentence surprisal significantly influences reading speed across multiple languages. Lower surprisal, indicating higher predictability, enables readers to process sentences more rapidly without compromising comprehension. In contrast, higher sentence surprisal slows reading, signaling the additional cognitive resources required for understanding less predictable content. The impact of sentence surprisal on reading aligns closely with the influence of word surprisal on the reading process (Goodkind & Bicknell, 2018; Wilcox et al., 2023). Prediction occurs not only at the level of individual words but also at the level of entire sentences in discourse (Ferreira & Chantavarin, 2018; Kuperberg & Jaeger, 2016; Van Berkum, 2008). Prediction could be an important mechanism underlying human language comprehension and processing, which makes predictions about the likely content of the upcoming input.

Recent studies highlight the importance of global semantic relatedness at the discourse level in language comprehension and processing (e.g., Carter & Hoffman, 2024; Lewis et al., 2017). Rooted in discourse coherence theories (Gernsbacher, 1997; Kintsch, 1998), this view holds that comprehension improves when new information connects meaningfully to earlier content. Sentences closely tied to prior context are processed more quickly, while semantically distant ones slow processing. Our proposed metric of sentence relevance captures how well a sentence integrates with surrounding discourse, reflecting the role of semantic coherence in supporting comprehension and reading speed. It quantifies how closely a sentence aligns with the meaning of its neighbors, drawing on memory‐based models that emphasize contextual integration (e.g., Lewis & Vasishth, 2005; Traxler, 2011). Unlike surprisal, which reflects unpredictability, semantic relevance indexes the ease of retrieving and linking prior context.

This construct is grounded in cognitive principles of discourse comprehension. Strong semantic ties improve retention and processing efficiency, especially when local context—typically the one or two preceding sentences—is considered (Rayner, Chace, Slattery, & Ashby, 2006; Smith & Levy, 2013). Working memory constraints and recency effects also shape how prior context influences processing, prompting differential weighting of contextual sentences. These factors collectively guide our computation of semantic relevance, capturing key mechanisms of coherent language processing.

Specifically, sentences with greater contextual relevance are read more quickly and with less cognitive effort, as they align somewhat with readers' expectations. Conversely, less relevant sentences demand greater cognitive resources, reflected in slower reading speeds. The impact of sentence relevance on reading closely resembles the effect of word semantic relevance on reading (Sun et al., 2023; Sun & Liu, 2025). There are several possible reasons why relevant sentences tend to be processed more quickly. According to the research on discourse structure, relevant sentences can easily create discourse coherence. One important factor is the activation of mental representations related to the topic or theme of the text (Traxler, 2011). When a sentence is relevant to the context, it could activate mental representations through memorizing the context, making it easier for readers to process the sentence. This activation can lead to faster reading speed as readers are able to effectively activate the memory of the context and quickly integrate it into their mental representation of the text.

Other predictors also modulate the relationship between reading speed and comprehension. For instance, as shown in Fig. 2, sentences containing longer words (i.e., “mean word length”) consistently result in slower reading speeds across languages, reflecting the increased processing demands for comprehension. In contrast, sentences with more frequent words (i.e., “mean word frequency”) tend to be read more quickly while maintaining comprehension. These effects are remarkably consistent across different languages, suggesting fundamental aspects of cognitive processing that transcend individual languages.

### 5.3 Integrating sentence metrics and cognitive mechanisms

The interplay between sentence surprisal, semantic relevance, and cognitive mechanisms such as prediction and memory provides a unique understanding of sentence processing. Sentence surprisal captures the unpredictability of linguistic input, while semantic relevance reflects the ease with which new information integrates into the preceding context. These metrics could interpret how readers navigate the complexities of discourse.

Our findings suggest that prediction‐based mechanisms, as reflected by sentence surprisal, enable readers to anticipate upcoming information, facilitating smoother integration and faster reading speeds. At the same time, memory‐based mechanisms, influenced by semantic relevance, support the retrieval and alignment of prior context with new input. Semantic relevance aligns with memory‐based theories by quantifying how easily a sentence integrates with its discourse context. The memory‐aware weighting reflects the recency effect in working memory, where closer sentences provide stronger contextual cues, reducing cognitive load and accelerating reading speed. This complements the predictive role of sentence surprisal, supporting a dual‐mechanism framework where expectation and memory jointly shape sentence processing. These mechanisms interact dynamically (Futrell et al., 2020; Ryskin & Nieuwland, 2023) (shown in the last row of Table 2), with greater predictability and relevance yielding more efficient comprehension and processing.

Our findings that both sentence surprisal and semantic relevance predict reading speed also suggest a *dual‐mechanism framework* for sentence processing. The strong performance of sentence surprisal supports expectation‐based theories of language processing, indicating that readers form predictions not merely at the word level but also at the sentence level. Meanwhile, the complementary contribution of semantic relevance aligns with memory‐based processing theories, suggesting that sentence comprehension involves actively maintaining and integrating semantic information across sentence boundaries.

### 5.4 Limitations and future work

Several limitations of our current approach deserve attention. First, our study focuses on reading speed as a proxy for sentence comprehension. While reading speed is a useful indicator of processing difficulty and efficiency, it does not capture the full spectrum of comprehension processes. For instance, deeper‐level understanding, such as inference making, semantic integration, or pragmatic reasoning, may not be reflected in reading times alone. Future work could combine our sentence‐level metrics with more direct behavioral or neurocognitive measures, such as comprehension questions, recall, or neural responses, to more comprehensively assess language understanding.

Second, our current methods treat sentences as holistic units without explicitly considering their internal syntactic or compositional structure. This raises the possibility of a confound between sentence‐level metrics (such as sentence surprisal) and the cumulative influence of word‐level predictability. Some aspects of sentence processing may stem from syntactic integration or hierarchical composition, which are not fully captured by the current surprisal and relevance metrics.

This limitation intersects with a broader theoretical debate in psycholinguistics regarding the architecture of language processing. Specifically, it remains an open question whether language is processed in a strictly serial manner or through distributed mechanisms. Models such as EZ‐Reader (a serial model; Rayner, 1998) and SWIFT (a distributed‐processing model; Engbert, Nuthmann, Richter, & Kliegl, 2005) offer contrasting views. While our findings are broadly compatible with distributed processing accounts, given the influence of both predictive (surprisal‐based) and memory‐based (relevance‐based) mechanisms, we emphasize that our goal is empirical rather than adjudicative. A definitive comparison between processing architectures requires experimental paradigms that go beyond reading speed and engage with temporal dynamics more directly.

Furthermore, the distinction between word‐level and sentence‐level predictability warrants further empirical clarification. In typical corpora, sentence‐level predictability often covaries with the predictability of individual words, raising a potential confound between compositional structure and cumulative lexical predictability. This makes it difficult to isolate the unique contribution of sentence‐level information to processing difficulty. One promising direction for future research is to design experimental stimuli in which sentences exhibit high‐level coherence while containing individually unpredictable words or vice versa. Such designs would provide a stronger test of whether sentence‐level surprisal offers explanatory power beyond what is captured by word‐level predictors alone.

Finally, although our results generalize across 13 languages, we do not account for typological differences such as morphological richness or syntactic flexibility, which may moderate the observed effects. Future work could explore how specific linguistic features interact with surprisal and relevance, and whether different languages rely more heavily on predictive versus memory‐based mechanisms.

## 6 Conclusion

This study presented two sentence‐level metrics for predicting human processing of sentences as a whole. Using multilingual LLMs, the methods of computing sentence surprisal worked well, and the “memory‐aware” method allowed for computing contextual information for sentence‐level semantic relevance. The results show that both sentence surprisal and sentence relevance were highly capable of predicting human sentence reading speed. All of these methods also exhibited strong generalization capabilities across languages. The findings showed that these sentence‐level metrics are informative features for modeling entire sentence processing by humans. We believe that our sentence‐level metrics may also serve as predictors of other sentence‐level cognitive and neural measures in humans. Our work highlights the potential of integrating LLMs with cognitive models to better explain and predict human language comprehension and processing and to inspire the development of more effective artificial general intelligence systems.

## Supporting information

Figure S1: The parafoveal‐on‐foveal effects in reading (from Sakurai (2023))
Figure S2: The memory capability and weights adopted in the memory‐aware approach
Figure S3: The partial effects of the primary predictors on reading speed across 13 languages.
Table S1: Variable importances of sentence surprisal and sentence relevance according to language‐specific GAMMs for reading speed
Figure S4: Pearson correlation between sentence‐level surprisal (computed by m‐BERT and chain rule) and sentence‐level semantic relevance (computed based on m‐BERT) in each language.

## Data Availability

The code and data in this study are available at https://github.com/fivehills/Multilingual_sentence‐surp_and_sentrev

## Notes

1 To facilitate a consistent comparison of sentence surprisal, we used the same language models (i.e., m‐BERT and mGPT). However, sentence embeddings generated by models such as sentence‐transformers/paraphrase‐multilingual‐MiniLM‐L12‐v2 can more effectively capture sentence‐level information. As a result, the performance in measuring sentence relevance has improved significantly. We have provided the Python script for computing sentence relevance using Sentence Transformers.

2 “Mean word length” refers to the average length of all words within a sentence (i.e., *the sum of length of all words/word number*), while “mean word frequency” represents the average of the sum of normalized frequencies for all words within a sentence (i.e., *the sum of normalized word frequencies/word number*). The word frequency information across languages is obtained from https://opus.nlpl.eu/OpenSubtitles/corpus/version/OpenSubtitles (Lison & Tiedemann, 2016).
