# Supplementary material for: Computational Sentence‐Level Metrics of Reading Speed and Its Ramifications for Sentence Comprehension
Source: Cogn Sci. 2025 Jul 22;49(7):e70092. doi: 10.1111/cogs.70092 (PMC12281087; doi:10.1111/cogs.70092)
Supplement: Supplementary file 1 — Figure S1: The parafoveal‐on‐foveal effects in reading (from Sakurai (2023)) Figure S2: The memory capability and weights adopted in the memory‐aware approach Figure S3: The partial effects of the primary predictors on reading speed across 13 languages. Table S1: Variable importances of sentence surprisal and sentence relevance according to language‐specific GAMMs for reading speed Figure S4: Pearson correlation between sentence‐level surprisal (computed by m‐BERT and chain rule) and sentence‐level semantic relevance (computed based on m‐BERT) in each language. [file COGS-49-e70092-s001.pdf]

The **The Supplementary Material (SM)** of "Computational Sentence-level Metrics of Reading Speed and its Ramifications for Sentence Comprehension"

Kun Sun
Tongji University, China/
University of Tübingen, Germany
kunsun@tongji.edu.cn

Rong Wang
University of Tübingen, Germany/
University of Stuttgart, Germany
rong.wang@uni-tuebingen.de

A. Differences between reading speed and fixation duration

In reading research, reading speed and fixation duration for individual words represent distinct but related aspects of how humans process language. The following specifies these differences.

Reading speed (or average reading speed, or reading rate) is typically measured in words per minute or second (i.e., `wpm`) and reflects the overall rate at which a person reads a passage of text or a sentence. Reading speed is usually employing text or individual sentence as the unit of interest. This metric gives a general indication of reading efficiency or fluency. It measures the reader's ability to comprehend and process text or sentence over a given period. This can vary significantly depending on the reader's skill, the text's complexity, and the reading purpose (e.g., reading for pleasure vs. critical reading). Reading speed encompasses the entire reading process, including both the time spent on fixations (when the eyes stop on a word) and saccades (the rapid movements between fixations), as well as regressions (backward movements to re-read text) and pauses for comprehension. Reading speed has been extensively and intensively explored (Jackson and McClelland, 1979; Herman, 1985; Carver, 1990; Rayner et al., 2010).

Fixation duration for individual words (here "total fixation duration")

refers to the total length of time the eyes remain stationary during fixations on a word while reading. These durations are measured in milliseconds with an eye-tracker and can indicate the cognitive effort required to process the word. Fixation duration on individual words can reveal how word characteristics (such as length, frequency, and predictability) and contextual difficulty affect reading. Longer fixations tend to suggest that a word is harder to recognize or comprehend within its context. Fixation durations vary across words within a text and among different readers. They provide insight into the moment-by-moment cognitive processes involved in lexical reading, such as lexical access, parsing, and integration of information.

Reading speed offers a macro-level view of reading behavior, indicating overall efficiency or difficulty at the sentence or text level. In contrast, fixation duration provides a micro-level perspective, revealing the cognitive processing time for individual words. While both metrics can be affected by the text's difficulty and the reader's proficiency, reading speed could be also influenced by broader factors like braoder context and the reader's strategy or purpose. Fixation duration is more directly related to immediate cognitive demands for word-specific characteristics.

B. Computing sentence surprisal

This section details how to compute sentence-level surprisal using the three methods: chain rule (CR), negative log-likelihood (NLL) and next sentence prediction (NSP). The first two methods were applied in either `m-BERT` or `mGPT`, and the third method was merely implemented in `m-BERT`.

The following provides a detailed account of how to apply **chain rule (CR)** to compute sentence surprisal . First, we tokenized sentence into various tokens: $S = [t_1, t_2, \ldots, t_n]$. Probability of a 0token t_i in the sentence S given its left context C in a text and preceding tokens can be represented as: $P(t_i|C, t_1, t_2, \ldots, t_{i-1})$. ($S$ = sentence, t = token, C = context). Based on this, we can calculate the probability of each token prior to its left context. After obtaining the probability of each word, we computed the joint probability of the entire sentence in Equation (2): S given its context C, $P(S|C) = P(t_1|C) \cdot P(t_2|C, t_1) \cdot P(t_3|C, t_1, t_2) \cdot \ldots \cdot P(t_n|C, t_1, t_2, \ldots, t_{n-1})$ (2), further getting sentence surprisal, $-log(P(S|C))$. The equation is an application of the chain rule of probability to sequences (like sentences). It captures the idea that the probability of a sentence given a context can be decomposed into the product of conditional probabilities of its individual tokens.

The following explains why CR works. When we have a sequence of events or tokens, the chain rule can be extended. For a sequence of three events t_1, t_2, and t_3: $P(t_1, t_2, t_3) = P(t_1) \times P(t_2|t_1) \times P(t_3|t_1, t_2)$. In the case of natural language, sentences often have a context. The context can be prior knowledge, a preceding sentence, or any other relevant information. Given a context (C), the probability of a sequence changes (S): $P(S|C) = P(t_1|C) \times P(t_2|C, t_1) \times P(t_3|C, t_1, t_2) \times \ldots \times P(t_n|C, t_1, t_2, ..., t_{n-1})$ (i.e., the *multiplication* of probabilities of each token given the left context within a sentence). The method of computing sentence probability is consistent with the principles of probability theory and the way sequence modeling is approached in the context of NLP (natural language processing).

Moreover, using **Negative Log-Likelihood (NLL)**, we can also compute the surprisal of a sentence conditioned on its preceding textual content. The specific procedure is detailed as follows: the text is tokenized into a sequence of token IDs, converted into a `PyTorch` tensor, and processed through the model to determine the NLL. NLL is used as a loss function to measure how well the model's predictions align with the actual data. For a given sequence of tokens, NLL is a measure of how surprised the model is by the actual sequence. There are two steps in calculation: Step one is to *sum up* these NLLs for the words in the sentence: $\text{NLL}(S\Big|C) = \sum \log(P(t_i|t_1, t_2, ..., t_{i-1})$ (t_i is the word in the target sentence "S", and t_1 , t_2 , ..., t_{i-1} include both the context and the preceding words in the sentence) Step two is to convert NLL to probability, as shown in Equation (3): $P(S|C) = e^{-\text{NLL}(S|C)} \quad (3)$. The term $e^{-\text{NLL}}$ represents an exponential function with the negative Natural Log Loss (NLL) of S given C as the exponent.

We elaborate on the *differences* between applying Chain Rule (**CR**) and Negative Log Likelihood (**NLL**) within the contexts of `BERT` and `GPT`, detailing these distinctions for each model respectively. Within the context of autoregressive language models (LMs) like `GPT`, the concepts of the CR for probability and NLL indeed converge when computing sentence surprisal. The CR decomposes the probability of a sequence into the product of conditional probabilities. This method aligns with how autoregressive LMs generate text, predicting each token based on the previous ones. NLL is often used as a loss function in training these models, calculated by taking the negative logarithm of the probability of the observed data under the model. For sentence, NLL would be the *sum* of the negative logs of these conditional probabilities. When we take the logarithm of the product of conditional probabilities (as per the CR), it becomes a *multiplication* of these probabilities. The math equations for CR and NLL introduced at the start of this

section are applicable to the GPT case.

Although the chain rule of probability naturally applies to autoregressive language models that predict tokens in a strictly left-to-right manner, BERT's bidirectional architecture presents a challenge for directly computing sentence-level probabilities. BERT is trained through masked language modeling (MLM), which predicts masked tokens based on their surrounding context, incorporating both preceding and succeeding words. Therefore, it does not model token sequences autoregressively and does not natively support the standard chain rule formulation:

$$P(t_1, t_2, \ldots, t_n) = \prod_{i=1}^{n} P(t_i \mid t_1, \ldots, t_{i-1})$$

To approximate sentence-level surprisal with BERT, we implement a masking-based strategy that mimics the chain rule without requiring unidirectional modeling. For each token t_i in a sentence $S = \{t_1, t_2, \ldots, t_n\}$, we mask it individually and use BERT to estimate its probability given the remaining tokens in the sentence and any preceding context C. This provides a pseudo-conditional probability $P(t_i \mid S_{-i}, C)$, where S_{-i} is the sentence with the i-th token masked.

The total sentence-level probability is approximated as the product of these token-level probabilities:

$$P(S \mid C) \approx \prod_{i=1}^{n} P(t_i \mid S_{-i}, C)$$

and the corresponding surprisal is calculated as:

$$\text{Surprisal}(S \mid C) = -\log P(S \mid C) = -\sum_{i=1}^{n} \log P(t_i \mid S_{-i}, C)$$

This approach employs BERT's bidirectional prediction capacity to approximate a form of chain-rule-based surprisal, despite the model's non-autoregressive design. It enables the estimation of how predictable a sentence is, given both its internal structure and external context.

We then focus on NLL in BERT. We compute sentence surprisal using BERT based on negative log-likelihood (NLL) estimated via pseudo-likelihood masking. While NLL is a general concept used in machine learning to quantify model prediction error, its application to masked language models like BERT requires a specific adaptation. In our implementation, we adopt a

pseudo-likelihood approach: for each token in a target sentence, we mask it individually and use `BERT` to predict the masked token given all other tokens in the sentence, along with any preceding context. This takes advantage of `BERT`'s bidirectional attention, allowing each token to be predicted from both left and right context.

Formally, the sentence-level surprisal is computed as:

$$\text{NLL}(S \mid C) = -\sum_{i=1}^{n} \log P(t_i \mid S_{-i}, C)$$

where $S = \{t_1, t_2, ..., t_n\}$ is the target sentence, S_{-i} denotes the sentence with the i-th token masked, and C represents the preceding context (if any). The total NLL is obtained by summing over the negative log-probabilities of all tokens, thus providing a scalar measure of sentence surprisal. This token-by-token masking strategy is standard in evaluating pseudo-likelihood for masked language models (Salazar et al., 2019), and enables us to estimate how predictable each sentence is in its given context, without requiring an autoregressive model.

In addition to adopting the CR and NLL, we could employ the *"next sentence prediction (NSP)"* mechanism in `BERT` to compute sentence surprisal. To compute the probability that one sentence follows another using `BERT`'s NSP, begin by preparing the input. Combine the two sentences, placing a special [CLS] token at the start and a [SEP] token in between them. After tokenizing this combined sequence with `BERT`'s tokenizer, pass it through the `BERT` model. `BERT` uses the representation of the [CLS] token, which encapsulates information about the entire input, to predict the relationship between the two sentences. The model outputs two probabilities: "IsNext" and "NotNext". The "IsNext" probability indicates the likelihood that the second sentence logically follows the first. By examining this probability, one can gauge how likely the model perceives the given sentence order to be. The above description can be summarized as the following two equations. Probability of sentence B being the next sentence given the left context A is formalized as (5):

$$P(B|A) = \text{softmax}(w_2^T[\text{CLS}] + b_2) \qquad (5)$$

where `[CLS]` denotes the representation of the first sentence obtained from the final layer of the `BERT` model, and the `softmax` function computes the probability distribution over all possible next sentences. w_2 and b_2 are learnable weight vector and bias term, respectively. And then we can obtain the sentence surprisal. However, NSP in `BERT` is a binary classifier, that

is, the model determines if a sentence logically follows a given sentence or not. This is a simplification of the rich structure and semantics in natural language. The use of NSP may raise the question about the interpretability of `BERT`'s estimated probabilities for subsequent sentences, especially when viewed from a cognitive modeling standpoint. The reason for this is that we probably argue that as humans read, they probably take more than two sentences as the context window. Despite this, we still need to apply the statistical analysis to test whether such metrics are useful or not.

After providing a detailed account of how sentence surprisal is computed, the following section compares the main *differences between word surprisal and sentence surprisal.* These comparisons can offer a deeper understanding of the comprehensive nature of sentence surprisal.

First, the primary distinction between word surprisal and sentence surprisal lies in their unit of analysis and predictive scope. Word surprisal is computed for individual words within a sentence, mostly relying on the preceding words in the same sentence to estimate the probability of the next word $\boxed{\text{word} \quad \text{surprisal = -log(P(word}|\text{left} \quad \text{context)}}$. This metric is commonly used to explain word-level fixation measures, such as gaze duration or first fixation duration during reading, for each word in a sentence. In contrast, sentence surprisal considers entire sentences as the unit of analysis. It incorporates broader discourse context, often extending to the two or three preceding sentences, to predict the overall likelihood of the current sentence $\boxed{\text{sentence} \quad \text{surprisal = -log(P(sentence} \mid \text{left} \quad \text{context))}}$. While we use token/word-level decomposition as a computational necessity by employing methods like negative log-likelihood (NLL) or the chain rule to compute sentence surprisal, the significant distinction lies in how our approach calculates $P(\text{sentence} \mid \text{left} \quad \text{context})$ as a unified probability for the entire sentence. Rather than treating token-level probabilities as individual predictors, they are integrated to capture the joint likelihood of the complete sentence structure. This approach enables us to model sentence-level phenomena that arise from the interaction of multiple tokens and words. Additionally, sentence surprisal is typically employed to predict sentence-level measures, such as reading speed for the entire sentence.

Although both word surprisal and sentence surprisal are derived from token probabilities in probabilistic models, their computational approaches differ significantly. Word surprisal is calculated using a left-to-right method within a single sentence, considering the preceding words as context. Sentence surprisal, whether computed using the chain rule, negative log-likelihood (NLL), or `BERT`-based NSP, differs from the left-to-right method used for

calculating word surprisal. Any of these methods incorporates a broader discourse context by accounting for dependencies between sentences. This enables sentence surprisal to capture higher-level patterns such as coherence and discourse structure, which are beyond the scope of word-level computations. These computational differences demonstrate the complementary roles of word and sentence surprisal in modeling language processing.

Second, direct statistical comparisons between word surprisal and sentence surprisal are not feasible due to differences in response variables and analytical granularity. Word surprisal is associated with word-level fixation measures like gaze duration, whereas sentence surprisal predicts sentence-level measures, such as reading speed (for text or sentence). These distinct response variables reflect the divergent focus of the two measures. Additionally, word surprisal provides a fine-grained analysis at the token/word level, while sentence surprisal aggregates data across entire sentences, resulting in a coarser analytical perspective.

Overall, the distinctions in unit, response variable, granularity, and computational scope/method make direct comparisons between word surprisal and sentence surprisal impractical in statistical models. However, these metrics serve complementary purposes, providing insights at different levels of linguistic analysis. Word surprisal is particularly suited for explaining word-level fixation data, but sentence surprisal offers a more holistic perspective on sentence-level comprehension within broader discourse contexts. Together, they contribute to a comprehensive understanding of human language processing.

C. BERT approximates human reading behavior

Some researchers have underestimated `BERT`'s potential in calculating word probabilities, arguing that the use of `BERT` (based on masked LMs) is hard to justify within the realm of cognitive modeling. Their concern stems from the fact that a masked language model like `m-BERT` can consider context from both directions, including rightward context, which could not be typically how human comprehension works. However, `BERT` could be potentially able to estimate word probability because it can be line with human reading behavior. The following elaborates on this point from both theoretical and empirical perspectives.

From a theoretical perspective, surprisal in linguistics refers to the degree to which an item of linguistic unit is predictable within a given context. It quantifies the degree of unexpectedness a linguistic unit carries when encountered in a particular context. While surprisal is often associated with left-to-right language models that predict the next linguistic unit based on prior context, it is a broader and more flexible concept. Surprisal does not inherently depend on sequential directionality; rather, it captures the information content or processing difficulty associated with a linguistic unit, regardless of the specific predictive framework.

Although next-unit prediction remains a common application, surprisal can also be derived from models that use richer, non-directional contextual information. Bidirectional models such as `BERT`, for example, incorporate both preceding and following words to represent context. This enables a comprehensive estimation of surprisal that aligns with how humans process language, by integrating information from the entire sentence or discourse context. In this way, computing surprisal with bidirectional models reflects a cognitively plausible approach that extends beyond traditional sequential prediction.

We then discuss this point from the empirical perspective. Those familiar with eye-tracking in reading recognize the preview benefit (i.e., parafoveal-on-foveal effects, "PoF" for short). Parafoveal-on-foveal effects in reading refer to the influence of information from the words located in the parafoveal region (the area immediately surrounding the fixated word) on the processing of the currently fixated word (the foveal word) (Kennedy and Pynte, 2005; Kliegl et al., 2007; Schotter et al., 2012; Hohenstein and Kliegl, 2014). As the PoF effect takes place in the context of reading and visual processing, the visual perception of words not directly fixated upon (parafoveal words) influences the processing of the word currently fixated on (the foveal word).

Put it simply, when we read naturalistic texts, our eyes do not always move smoothly across the text but make quick, jerky movements known as saccades, interspersed with brief pauses called fixations. During these fixations, the eye directly focuses on a small area of text. This area, where visual acuity is highest, is known as the foveal region. Surrounding the foveal region is the parafoveal region, where vision is not as sharp but still capable of processing some information about the text, such as word length or initial letters. The PoF effect refers to how information from the parafoveal region can pre-activate or facilitate the processing of the word once it becomes the focus of the next fixation (moves into the foveal region), thereby affecting reading speed and comprehension. For example, if the parafoveal word is

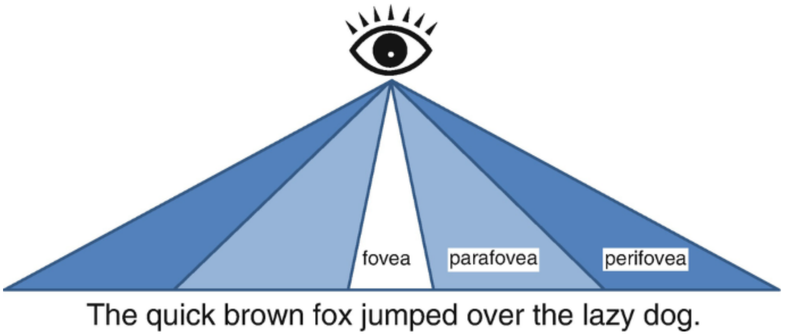

Figure S1: The parafoveal-on-foveal effects in reading (from Sakurai (2023))

semantically or syntactically related to the foveal word, it might speed up the recognition and processing of the foveal word when the eye moves to it (Pan et al., 2021; Schotter et al., 2012; White et al., 2011). In short, processing the target word actually is likely to be involved in incorporating the information on the next one or two ore more words (n+1, or n+2 word in literature) through previewing, as shown in Figure S1.

Relevant research shows that reading times are shorter for a target word when it matches the preview word, compared to when they are different. This suggests that the preview word is processed using parafoveal vision. `BERT` mirrors this process to some extent because it can incorporate information from subsequent words, approximating how human parafoveal vision previews words to the right of the fixation. In this sense, `BERT` aligns with human reading behaviors to a greater degree. The surprisal values calculated by `BERT`, reflecting real-world reading dynamics, potentially provide precise predictions of word reading times. The criticism that `BERT` generates only pseudo-surprisal overlooks its relevance to real-world human reading behaviors. On the other hand, surprisal values computed by `GPT` do not account for the previewing aspect of human reading. Despite this, surprisal values from both `GPT` and `BERT` can, from different theoretical perspectives, offer insights into human reading behaviors. Nevertheless, statistical analysis is needed to determine which model's surprisal predictions align more closely with observed reading data.

D. Memory-aware approach and its justifications

This section provides a comprehensive guide on calculating sentence relevance using the "memory-aware" approach, executed in a two-step strategy. This section also details the justifications of "memory-aware" method from linguistic, cognitive and mathematical perspectives.

The sliding window includes four sentences (t, 2, 1, and n1), as shown in the Panel B of Fig. 1 in the main text. 't' represents the target sentence, and 'n1' for the next sentence. Moreover, '1' refers to the immediately preceding sentence, while '2' denotes the sentence before that. We aim to compute how the target sentence (t) is semantically related with the other three sentences (2, 1, and n1 form the context window).

The initial step involves generating sentence embeddings using `m-BERT` or `mGPT`, followed by computing the similarity between two sentences based on these embeddings. The subsequent step entails applying the "memory-aware" approach to manage multiple similarity values across several sentences within a window stack.

First, we need to generate embeddings for each sentence in this window by employing `BERT` or `GPT`. After obtaining sentence embedding for each sentence, we applied `cosine similarity` to compare the sentence embeddings generated by `m-BERT` or `mPGT`, and this practice is a common approach for computing semantic similarity for a sentence pair, formalized as Equation (6).

$$\text{similarity}(s, c) = \frac{e_s \cdot e_c}{\|e_s\| \|e_c\|} \qquad (6)$$

where s is the input sentence, c is the left context, e_s and e_c are their respective sentence embeddings obtained using mean pooling with `BERT`, $\cdot$ denotes dot product, and $\|\cdot\|$ denotes L2 norm. The second term is a modified form of the cosine similarity to account for the distance between the embeddings.

We employed two distinct multilingual LLMs (i.e., `m-BERT` and `mGPT`), noting subtle differences in their approaches to generating sentence embeddings. Regarding `m-BERT`, when a sentence is input into `BERT`, it is first tokenized and then produced with a special "`[CLS]`" token. After processing through `BERT`'s layers, the embedding corresponding to this "`[CLS]`" token is often used as the sentence embedding. The tokenized input sentence (S) is represented as `[CLS]`, t_1, t_2, $\ldots$, t_n. After processing through `BERT`, the output embeddings at the final layer for this sequence are $[E_{\text{CLS}}$, E_{t_1}, E_{t_2},

..., E_{t_n}]. Mean pooling involves calculating the average of the embeddings of all tokens in the sequence `[CLS]`.

Here is Equation (7) to compute a sentence (or a text) embedding using `m-BERT`.

$$e_s = \text{BERT}_{\text{pooler}}(s) = \frac{1}{n}\sum_{i=1}^{n} h_i \quad (7)$$

where s is the input sentence, and $\text{BERT}_{\text{pooler}}$ is a mean pooling layer that takes the output vectors h_i of all n tokens in the input sentence and computes their average to obtain the sentence representation e_s.

Alternatively, we used `mGPT` to compute sentence similarity for cross-validation. We still applied embedding-based method to do this. Specifically, `mGPT` was to obtain embeddings for each sentence. Still employing the mean pooling, we used the hidden states of the sentence to represent the embedding. *Cosine* similarity was to calculate the similarity between the two embeddings, which is similar to `BERT`-similarity computation.

The subsequent discussion highlights the distinctions between `m-BERT` and `mGPT` in generating sentence-based embeddings. First, `mGPT`, being primarily focused on generative tasks, does not utilize a special token like `[CLS]` for aggregating sentence meaning. `GPT` architecture is designed to predict the next token in a sequence based on the previous context, which inherently focuses on a unidirectional flow of information. The other difference is to use pooling for sentence embeddings. Specifically, to obtain sentence-level embeddings from `GPT`, one common method is to aggregate the hidden states (from the last layer) of all tokens in the output. Mean or max pooling can be applied to these token-level embeddings to create a single vector representing the entire sentence. This approach leverages the contextual information encoded by `GPT` in a sequential manner, albeit without the bidirectional context that `BERT` captures. In `BERT`, pooling is an alternative to using the "`[CLS]`" token embedding, offering a way to capture a distributed representation of sentence meaning. However, in `GPT`, pooling is a necessary step for sentence-level representation since the model lacks a mechanism like the `[CLS]` token for summarizing the text.

The similarity score between any two sentences in this window is calculated. Following this, four cosine similarity values are obtained for the sentences. Subsequently, we implemented the second step.

After completing the first step, we elaborated on applying weights to calculate sentence relevance. Upon acquiring the similarity values for sentences within the window stack, we proceeded to apply weights to these values. The

process involves aggregating the weighted similarity values to derive a final score, as detailed in the main text. This allows the memory-aware approach to capture contextual information and to explain the underlying mechanism of the weighting system.

As illustrated in the Panel B of Fig. 1 in the main text, in a window stack, sentences closer to the target sentence resemble the initial days in the forgetting curve, while more distant words resemble the latter days. To simulate human forgetting mechanism, we allocated larger weights to the closer sentences and smaller weights to the distant sentences and the similarity between human forgetting mechanism and attentional weights adopted in the current study. The weight values gradually decrease with the distance between the target sentence and the contextual sentences (see Equation (1) and the Panel B of Fig. 1 in the main text), similar to the forgetting curve (Loftus, 1985). The memory-aware approach could be linked to memory models in terms of how memory is decayed during the encoding of information, which subsequently affects how humans process sentences during reading. More importantly, the " memory-aware" approach is computational and fundamentally memory-based, facilitating memory storage, retrieval, and integration. This approach not only realizes the memory function but also incorporates the expectation effect.

Furthermore, when applying a 3-sentence window (the target sentence is not taken into account), similar to the fading of memories over three days, the average rate of memory decline can be estimated to average one-third per day. This fading initiates more abruptly and slows as time progresses. By modeling this, we can recalibrate the significance of sentences based on their distance from the target sentence, diminishing their value by a one-third. In this sense, sentences further from the target sentence receive lower weights compared to those in a uniform distribution. This leads to a gradual weighting scale, such as [1, 0.7, 0.3], and so forth, mirroring their relative proximity to the target sentence.

Moreover, when incorporating multiple sentences into a window for analysis, an appropriate window size may involve computing sentence-level metrics for several sentences in a text. A window size of 3–4 sentences is generally sufficient to compute metrics for the majority of sentences in the text. However, if the window is too large, the initial sentences may be excluded from computation, resulting in many sentences lacking their metrics. The three/four-sentence window emerged as the optimal balance, providing sufficient context for semantic integration while remaining cognitively plausible. This choice ensures that our approach captures meaningful discourse connec-

tions while adhering to cognitive and computational constraints, enhancing both interpretability and performance.

The Panel A of Figure S2 shows how the "memory-aware" approach we adopted simulates a short-term memory stack, mirroring how readers retain memory of previously encountered sentences and their meanings. The method for using various weights of semantic relevance between any two sentences is inspired by both the attention mechanism found in Transformers (Panel A in Figure S2) and the human process of forgetting (Panel B in Figure S2). The "memory-aware" approach can facilitate effective incorporation of contextual information and enabling it to achieve memory storage, retrieval, and integration.

Finally, as previously noted, the term "memory-aware" bears a resemblance to the function of attention in Transformers, primarily because our approach we proposed significantly enhances computational efficiency through the incorporation of contextual information. However, our "memory-aware" method does not incorporate any attention layers from Transformers. Each step in the computation of memory-aware metrics is transparent and interpretable.

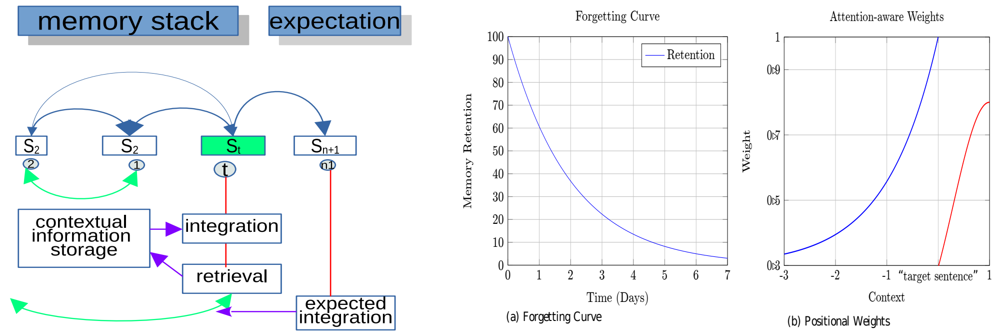

A. Memory capabilities of attention-aware approach

B. Weigths and forgetting curve

Figure S2: The memory capability and weights adopted in the memory-aware approach

The following section provides a detailed justification of the "memory-aware" approach from cognitive, linguistic, and mathematical perspectives. Each perspective highlights the theoretical and practical foundations of this methodology.

Cognitive perspective: The inclusion of the semantic relevance metric in our study is grounded in cognitive theories of human language processing, which emphasizes the integration of new information with prior context for

effective comprehension. Semantic relevance reflects how readers incorporate new sentences into their mental representation of a discourse. During natural reading, comprehension involves constructing and maintaining a coherent mental model, integrating incoming information with existing context, and identifying semantic relationships across sentence boundaries. This process requires cognitive resources (e.g., attention during reading) to be allocated in proportion to the semantic connectedness of discourse elements.

Memory-based processing theories further support this perspective, suggesting that previously encountered content remains partially activated in working memory, as shown in Figure S2. New information cues the retrieval of semantically related content, with the ease or difficulty of integration depending on the strength of these connections. When sentences are less connected to the active discourse context, processing demands increase, directly influencing reading speed and comprehension effort.

Linguistic perspective: Semantic relevance and word surprisal offer complementary insights into discourse comprehension by addressing distinct but interconnected mechanisms. Sentence surprisal captures forward-looking, expectation-based processing, enabling readers to predict upcoming content. Conversely, semantic relevance focuses on backward-looking integration, connecting new input with prior discourse.

Readers allocate attention based on the semantic relevance of incoming information, showing stronger integration effects for closely related content. This backward integration mirrors human cognitive strategies during discourse processing, where the influence of semantic relationships typically exhibits a distance-based decay.

Mathematical perspective: The "memory-aware" computation of semantic relevance employs a mathematically precise *convolution* framework to model the integration of semantic influences across sentences. Specifically, this convolution method operates on two functions: $f(x)$, which represents semantic similarity values between sentences, and $g(x)$, a weighting kernel $[1, 0.7, 0.3, 0.1]$ that accounts for distance-based decay. The convolution is expressed as:

$$(f * g)(x) = \int_{-\infty}^{\infty} f(t)g(x-t)\,dt$$

For our discrete case, the sliding-window formulation is:

$$h[t] = \sum_{k=0}^{3} f[t-k] \cdot g[k] \quad (8)$$

Here: - $f[t]$ denotes the semantic similarity value at position t. - $g[k]$ rep-

resents the weighting kernel $[1, 0.7, 0.3, 0.1]$. - $h[t]$ is the resulting integrated semantic relevance at position t.

This formulation systematically aggregates semantic contributions across adjacent positions, effectively capturing local contextual dependencies. The convolution framework aligns with essential aspects of human sentence processing, including the preservation of sequential dependencies, which reflects the natural order of discourse. It also incorporates distance-based weighting, mimicking the decay in semantic influence as the distance between words increases. Additionally, the framework supports contextual integration by combining information from neighboring sentences into a unified representation, and it facilitates local-to-global aggregation, which helps in developing a coherent understanding of discourse structure.

This convolution-based method holds significant potential for computing semantic-related metrics. We have completed another manuscript focusing on applying this method to enhance existing metrics (e.g., word surprisal, word frequency, semantic relevance) by integrating contextual effects, thereby improving predictions of human language processing.

Comprehensive integration : The robustness of the "memory-aware" method arises from its integration of cognitive, linguistic, and mathematical principles. By employing the convolution framework, this method achieves theoretical alignment with cognitive models of discourse processing while maintaining computational rigor. Practical benefits include efficient computation, scalability, and interpretability, which enhance its applicability across various discourse contexts.

In a broader context, the "memory-aware" approach we introduced could be applied effectively in either word level or sentence level. The approach and its resulting metrics have demonstrated effectiveness in predicting eye movements during reading across languages (Sun, 2023; Sun et al., 2023; Sun and Liu, 2025; Sun et al., 2024a), offering insights into language comprehension. Additionally, our investigations reveal that these metrics are highly effective in predicting phonetic and acoustic features in spontaneous speech data, encompassing dimensions such as speech duration, intonation, pitch rate, and other acoustic properties (Sun and Wang, 2024a; Jin et al., 2024). Spontaneous speech reflects the dynamic and adaptive nature of language production, further highlighting the versatility of these metrics. In other words, the "memory-aware" metrics for word could predict word processing in language comprehension and production. Moreover, the "memory-aware" metrics adopted similar methods could predict human visual processing, and it indicates that the approach and the resulting metrics could predict human

multi-information processing.

Moreover, "memory-aware" metrics we computed have shown the ability to predict and explain neural activity associated with the processing of naturalistic discourse. This includes both electroencephalography (EEG) and functional magnetic resonance imaging (fMRI) signals (Sun et al., 2024b; Sun and Wang, 2024b). By linking linguistic processing to neural responses, these metrics offer a powerful framework for bridging cognitive processes with their underlying neural mechanisms.

E. Statistical methods and comparison standards

To meet our goals of accurately predicting multilingual eye-tracking data, we utilized Generalized Additive Mixed Models (GAMMs)(Wood, 2017). GAMMs are effective in analyzing nonlinear effects and multiplicative interactions between variables, making them ideal for evaluating the predictability of semantic similarity. They are more flexible than traditional regression methods in modeling complex relationships between variables. Eye-tracking data is simpler to analyze statistically than EEG and fMRI data, which makes it an ideal choice for our study on naturalistic discourse reading. However, assessing model performance and comparing models can be challenging, and relying solely on correlations can be limiting. Fortunately, GAMMs are well-suited for comprehensive and precise assessments of model performance. We compared models using `AIC` (Akaike's Information Criterion) values, where a smaller value indicates a better model.

`AIC` or `BIC` (Bayesian Information Criterion) are both measures of model fit that balance goodness of fit with model complexity. Lower values of `AIC` or `BIC` indicate better model fit. However, `AIC` is a popular criterion for comparing GAMMs, and it has some advantages over other criteria. `AIC` is designed to balance the trade-off between model fit and model complexity, penalizing models with more parameters. This makes it useful for selecting models that provide a good balance between fit and complexity. `AIC` is also relatively easy to compute and widely used in statistical modeling.

Comparing two GAMM (or LMER, Linear Mixed Effects Models) fittings, where one is larger than the other (having all the parameters of the other model and some additional ones), the likelihood will always be higher for the larger model. This is because a larger model can fit the data better

by having more parameters. However, directly comparing loglikelihoods is not appropriate when models have different sizes. Both `AIC` and `BIC` address this issue by incorporating a penalty for the number of parameters. `AIC` and `BIC` have different principles for penalizing complexity. Generally, `BIC` penalizes complexity more strongly than `AIC`, tending to favor smaller models unless both approaches agree. In essence, `AIC` is preferable when the main goal is prediction quality, as a slightly larger model can still provide good predictions, while a too small model usually does not. On the other hand, `BIC` aims to identify a reasonably sized true model by prioritizing parsimony. `BIC` is often better in finding the true model, but it has a higher chance of selecting a model that is too small, which is not favorable for prediction. In practice, the true model is often not "small", but for reasons such as interpretability, smaller models are sometimes preferred even if they have slightly worse prediction performance, in which case `BIC` may be preferred. In summary, from the perspective of `AIC`, it is better to fit a slightly larger model than a too small one for improved prediction quality. However, from the perspective of `BIC`, both excessively large and excessively small models are equally undesirable (Vrieze, 2012).

Moreover, the developer of `R` package on GAMM ("mgcv") used AIC to make model comparison (Wood et al., 2016; Wood, 2020). `AIC` has also been mostly taken to understand model performance in psycholinguistic research (Wieling, 2018; Baayen and Linke, 2020 and the relevant studies) if GAMM or generalized mixed-effect models are employed.

In the studies conducted by Wilcox et al. (2020) and Oh and Schuler (2023), the relationship between model perplexity and Δ`LogLik` (log-likelihood) was utilized to analyze the perceptual competence of surprisal generated by different LMs. Their objective was to determine which LMs were capable of generating more powerful surprisal based on various corpora. In contrast, the current study aims to assess the predictive performance of our algorithms.

We should point out some potential issues when using Δ`LogLik` as a criterion. Wilcox et al. (2020) used GAMMs for their analysis but did not incorporate any *random variables* in these models. Consequently, confirming the optimality of these GAMMs becomes challenging. On the other hand, Oh and Schuler (2023) mentioned that they employed LMER and included random effects. However, the LMERs used in Oh and Schuler (2023) did not include "word frequency" as a control predictor. It is well-established in psycholinguistics and cognitive science that both "word length" and "word frequency" are significant variables in predicting reading time. Including these two factors as *control predictors* is commonly practiced when studying

reading time. Therefore, when investigating reading time, it is advisable to include both "word length" and "word frequency" in GAMMs or LMERs. Meanwhile, an optimal model should also include random variables. Failure to do so may result in suboptimal GAMMs or LMERs for studying reading time. Moreover, The excessive application of heavy penalties (e.g., `k` in GAMMs) on the given metrics leads to overfitting in the mixed-effect models. For example, the heavy penalty on the specific metric results in the partial effect curve of this given metric becoming much steeper, and the `AIC` or likelihood in the model increasing remarkably. Conversely, removing such penalties eliminates these effects.

Additionally, Wilcox et al. (2020) used GAMM fittings including the metrics with high correlations. For example, many models include the word frequency of the target and surrounding words, which are also highly correlated. As a result, these predictors may introduce a high risk of multicollinearity in GAMMs (Dormann et al., 2013). A related case is found in Shain et al. (2024), where unigram surprisal was included as a control predictor to evaluate the effect of GPT-based word surprisal on reading time. However, since unigram and GPT-based surprisal are likely to be strongly correlated, this modeling choice may also introduce multicollinearity concerns. When collinearity occurs in GAMMs, it inflates standard errors, leading to unreliable or nonsignificant coefficient estimates, even for meaningful predictors. It also makes the model unstable and hard to interpret, as highly correlated variables (e.g., GPT-based surprisal and unigram surprisal) share variance, obscuring their individual contributions. This can distort effect directions and suppress true signals, resulting in misleading conclusions despite an overall good model fit. Therefore, identifying and minimizing collinearity is essential for drawing valid inferences about predictors in cognitive modeling.

Considering these factors, the GAMM models used in the present study include two *control predictors*: "the mean word length" for a sentence and the "mean word length" for a sentence. Additionally, *random variables*, "participants" in eye-tracking experiments, and "languages", were included. We used `fs` (i.e.,random smooths) to adjust the trend of a numeric predictor in a nonlinear way, which includes random intercept and random slope. In other words, we can allow the metrics of interest to explore their effects on various levels of random variables comprehensively. We employed `AIC` to compare the performance of different GAMMs. The baseline model excludes the *main predictor of interest* (sentence surprisal or sentence relevance) but retains the other elements in the full GAMM model. After fitting the regression models, we calculated the ΔAIC values for each GAMM model by subtracting

the `AIC` of the base GAMM model from that of a full GAMM model. A smaller `AIC` indicates better model performance. Similarly, a smaller ΔAIC also indicates better performance. Additioanlly, considering the best sentence surprisal and sentence relevance in each language, we used `t` tests to check wether ΔAIC values for two metrics have significant differences. The result shows that there is no significance difference. In other words, it is not easy to distinguish sentence surprisal or sentence relevance could predict reading speed better or not.

F. Performance of individual languages

The performance of an individual language is shown in Figure S3. There are two essential standards for interpreting the curve of partial effect in each plot. The first standard involves analyzing the curve's steepness. A steeper incline signifies a stronger correlation between the predictor and reading speed, whereas a gentler slope indicates a less pronounced effect. The second standard focuses on the curve's fluctuation around zero; a curve that hovers around zero suggests its impact is minimal. Understanding mixed-effect models is essential for appreciating the significance of these indicators, highlighting the `AIC` as the preferred tool for model comparison.

We established a significance threshold at a p-value below 0.01. As depicted in Figure S3,both sentence surprisal and sentence relevance exhibit significant effects across all 13 languages, although the effect of sentence surprisal is only marginal in Greek. For sentence surprisal, the trend in each language shows a negative relationship with reading speed, consistent with the overall pattern observed in Fig.2 of the main text. In contrast, sentence relevance tends to be positively related to reading speed, aligning with the general trend of this metric.

We compared the performance of sentence surprisal (m-GPT+CR) and sentence relevance (based on embeddings generated by m-BERT) in predicting reading speeds, with results summarized in Table S1. Sentence relevance outperforms sentence surprisal in 9 out of the 13 languages, with particularly large margins in Dutch, Italian, and German. In contrast, sentence surprisal shows stronger predictive power in 4 languages: English, Hebrew, Russian, and Turkish. In Spanish, both metrics yield identical ΔAIC values. Notably, sentence surprisal is not significant in Greek, as indicated by a positive ΔAIC.

These findings suggest that while both metrics contribute meaningfully

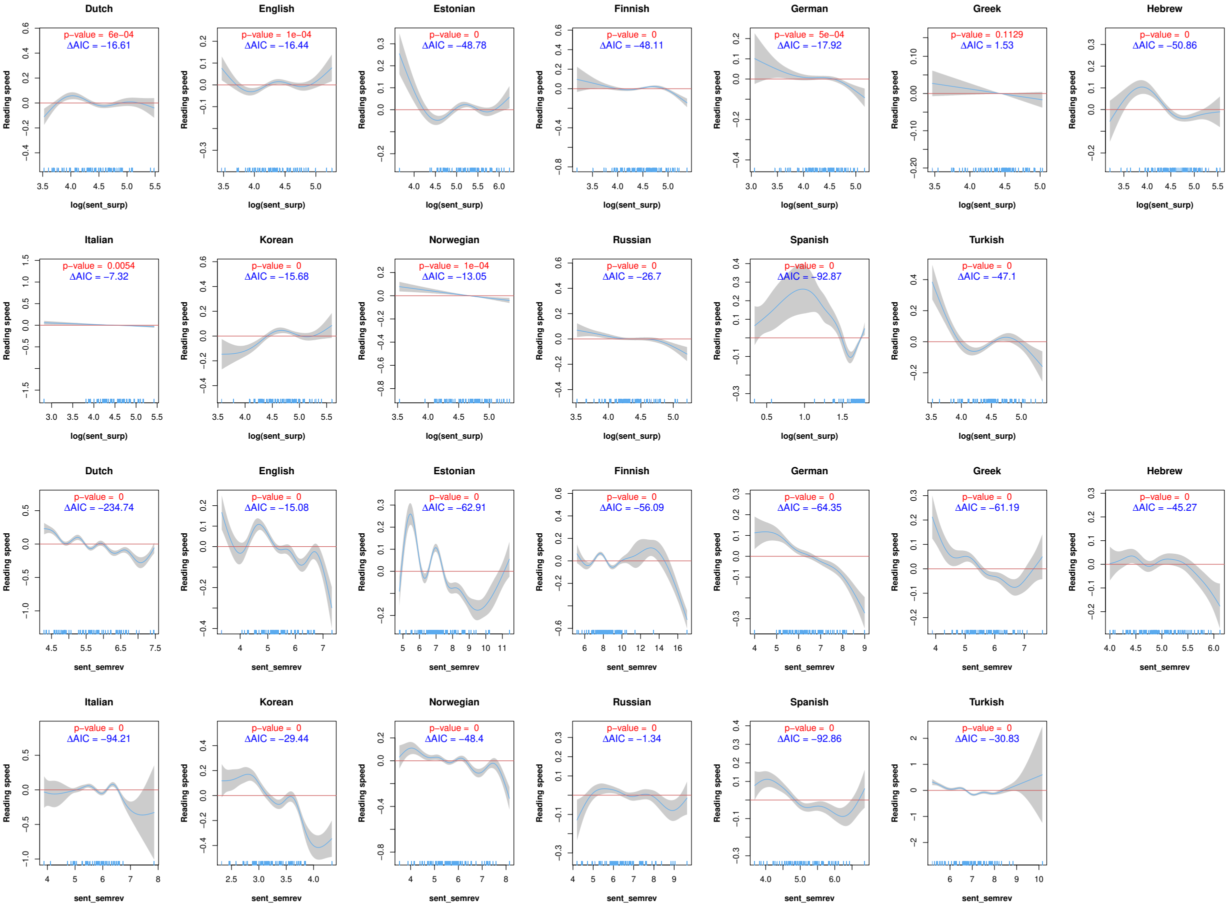

Figure S3: The partial effects of the primary predictors on reading speed across 13 languages. Note that the response variable, sentence surprisal and sentence relevance are transformed by logarithm (marked as "log_") to get closer distribution for better performance in GAMM fittings. The upper section of the diagram features sentence surprisal, while the lower portion is dedicated to sentence relevance.The *x-axis* signifies the computational metric, while the *y-axis* delineates the reading speed. To achieve a closer approximation to a normal distribution, and consequently improve the fitting, all metrics undergo a logarithmic transformation. Each curve visually articulates the correlation between a predictor variable and the response variable, namely reading speed. A steeper incline on these curves underscores a more robust impact between the predictor and reading speed, whereas gentler slopes imply a less pronounced effect. Moreover, when a curve fluctuates around zero, its effect vanishes. The information regarding p-values and ΔAIC is displayed at the top of each plot. The methodology for calculating ΔAIC for "sentence surprisal" and "sentence relevance" is detailed in the main text.

to modeling reading speed, sentence relevance generally offers stronger predictive utility across languages. This aligns with the broader pattern observed in the main analysis. As shown in Table 1S, sentence relevance consistently yields lower (i.e., better) Δ`AIC` scores in most languages, supporting its robustness as a cross-linguistic predictor of sentence-level processing difficulty.

Table S1: Variable importances of sentence surprisal and sentence relevance according to language-specific GAMMs for reading speed. Between parentheses: the ΔAIC, with a smaller value indicative of better performance.

Language	Predictors Comparison		
Dutch	sentence relevance (-293.73)	>	sentence surprisal (-16.62)
English	sentence relevance (-15.08)	<	sentence surprisal (-16.45)
Estonian	sentence relevance (-62.91)	>	sentence surprisal (-48.79)
Finnish	sentence relevance (-56.09)	>	sentence surprisal (-48.11)
German	sentence relevance (-64.35)	>	sentence surprsial (-17.92)
Greek	sentence relevance (-61.19)	>	sentence surprisal (1.01)
Hebrew	sentence relevance (-45.27)	<	sentence surprisal (-50.86)
Italian	sentence relevance (-94.21)	>	sentence surprisal (-7.32)
Korean	sentence relevance (-29.43)	>	sentence relevance (-15.68)
Norwegian	sentence relevance (-48.4)	>	sentence surprisal (-13.23)
Russian	sentence relevance (-1.34)	<	sentence surprisal(-26.37)
Spanish	sentence relevance (-92.86)	=	sentence surprisal (-92.86)
Turkish	sentence relevance (-30.86)	<	sentence surprisal (-47.83)

G. Correlation between sentence surprisal and sentence relevance

The overall `Pearson` correlation between sentence surprisal (computed using `m-BERT` with the chain rule) and sentence relevance (computed based on the sentence embedding derived from `m-BERT`) stands at ρ = **-0.054** (p-value <0.0001). The correlation value suggests that there is a weak correlation between the two metrics, and indicating that the two metrics are completely distinct. This relationship across the 13 languages is depicted in Figure S4. The observed correlations among the metrics for each language are notably minimal. Such low correlation scores demonstrate that the sentence surprisal and sentence relevance we calculated represent entirely distinct metrics.

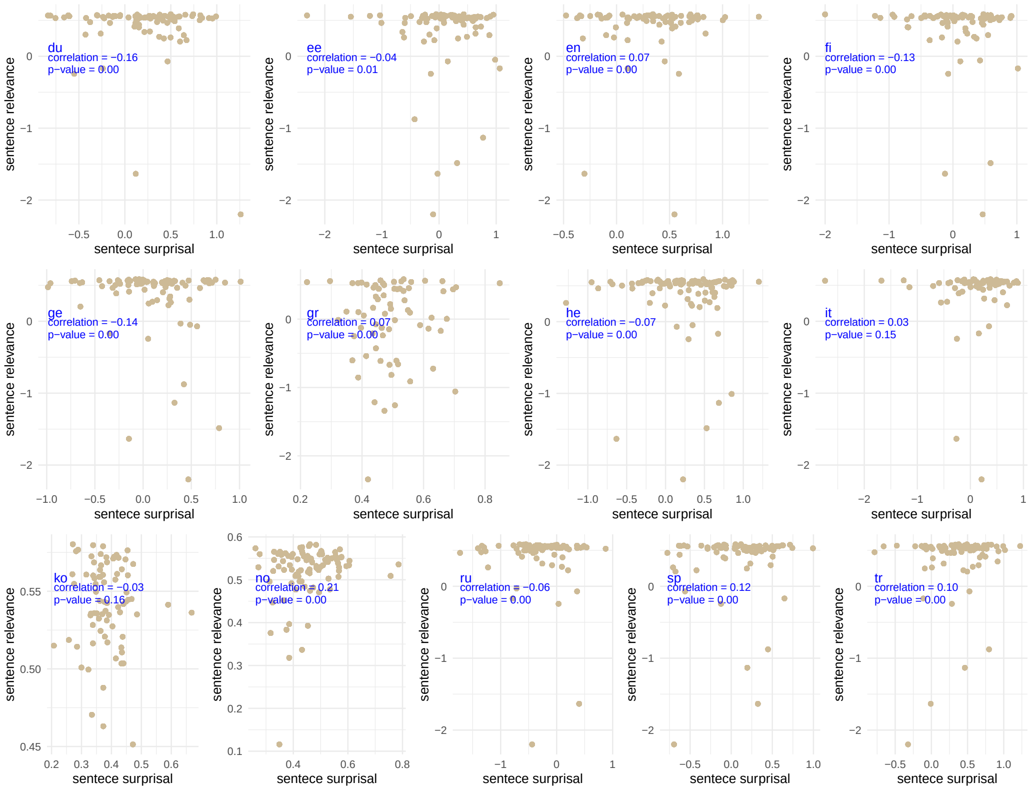

Figure S4: Pearson correlation between sentence-level surprisal (computed by `m-BERT` and chain rule) and sentence-level semantic relevance (computed based on `m-BERT`) in each language. Note: the abbreviations for these 13 melange are as follows. du = Dutch; ee = Estonian; en = English; fi = Finnish; ge = German; gr = Greek; he = Hebrew; it = Italian; ko = Korean; no = Norwegian; ru = Russian; sp = Spanish; tr = Turkish

References

Baayen, R. H. and Linke, M. (2020). An introduction to the generalized additive model. *A Practical Handbook of Corpus Linguistics*, pages 563–591.

Carver, R. P. (1990). *Reading rate: A review of research and theory.* Academic Press.

Dormann, C. F., Elith, J., Bacher, S., Buchmann, C., Carl, G., Carré, G., Marquéz, J. R. G., Gruber, B., Lafourcade, B., Leitão, P. J., et al. (2013). Collinearity: a review of methods to deal with it and a simulation study evaluating their performance. *Ecography*, 36(1):27–46.

Herman, P. A. (1985). The effect of repeated readings on reading rate, speech pauses, and word recognition accuracy. *Reading research quarterly*, pages 553–565.

Hohenstein, S. and Kliegl, R. (2014). Semantic preview benefit during reading. *Journal of Experimental Psychology: Learning, Memory, and Cognition*, 40(1):166.

Jackson, M. D. and McClelland, J. L. (1979). Processing determinants of reading speed. *Journal of Experimental Psychology: General*, 108(2):151.

Jin, X., Ernestus, M., and Baayen, R. H. (2024). A corpus-based investigation of pitch contours of monosyllabic words in conversational taiwan mandarin. *arXiv preprint arXiv:2409.07891.*

Kennedy, A. and Pynte, J. (2005). Parafoveal-on-foveal effects in normal reading. *Vision Research*, 45(2):153–168.

Kliegl, R., Risse, S., and Laubrock, J. (2007). Preview benefit and parafoveal-on-foveal effects from word n+ 2. *Journal of Experimental Psychology: Human Perception and Performance*, 33(5):1250.

Loftus, G. R. (1985). Evaluating forgetting curves. *Journal of Experimental Psychology: Learning, Memory, and Cognition*, 11(2):397.

Oh, B.-D. and Schuler, W. (2023). Why does surprisal from larger transformer-based language models provide a poorer fit to human reading times? *Transactions of the Association for Computational Linguistics*, 11:336–350.

Pan, Y., Frisson, S., and Jensen, O. (2021). Neural evidence for lexical parafoveal processing. *Nature Communications*, 12(1):5234.

Rayner, K., Slattery, T. J., and Bélanger, N. N. (2010). Eye movements, the perceptual span, and reading speed. *Psychonomic Bulletin & Review*, 17(6):834–839.

Sakurai, M. (2023). Parafovea. In *Encyclopedia of Color Science and Technology*, pages 1319–1326. Springer.

Salazar, J., Liang, D., Nguyen, T. Q., and Kirchhoff, K. (2019). Masked language model scoring. *arXiv preprint arXiv:1910.14659*.

Schotter, E. R., Angele, B., and Rayner, K. (2012). Parafoveal processing in reading. *Attention, Perception, & Psychophysics*, 74(1):5–35.

Shain, C., Meister, C., Pimentel, T., Cotterell, R., and Levy, R. (2024). Large-scale evidence for logarithmic effects of word predictability on reading time. *Proceedings of the National Academy of Sciences*, 121(10):e2307876121.

Sun, K. (2023). Optimizing predictive metrics for human language comprehension. *bioRxiv*, pages 2023–09.

Sun, K. and Liu, H. (2025). Attention-aware semantic relevance predicting chinese sentence reading. *Cognition*, 255:105991.

Sun, K., Wang, Q., and Lu, X. (2023). An interpretable measure of semantic similarity for predicting eye movements in reading. *Psychonomic Bulletin & Review*, 30(4):1227–1242.

Sun, K. and Wang, R. (2024a). Differential contributions of machine learning and statistical analysis to language and cognitive sciences. *arXiv preprint arXiv:2404.14052*.

Sun, K. and Wang, R. (2024b). The semantic relevance predicting fmri bold in narrative listening. *Manuscript*.

Sun, K., Wang, R., and Baayen, H. (2024a). Semantic integration predicts fixation durations across languages, independently of surprisal, word length, and word frequency. *Linguistics*.

Sun, K., Wang, R., and Nixon, J. (2024b). Semantic relevance predicting human neural activities during natural reading. *Manuscript*, pages 2024–09.

Vrieze, S. I. (2012). Model selection and psychological theory: a discussion of the differences between the akaike information criterion (aic) and the bayesian information criterion (bic). *Psychological Methods*, 17(2):228.

White, S. J., Warren, T., and Reichle, E. D. (2011). Parafoveal preview during reading: Effects of sentence position. *Journal of Experimental Psychology: Human Perception and Performance*, 37(4):1221.

Wieling, M. (2018). Analyzing dynamic phonetic data using generalized additive mixed modeling: A tutorial focusing on articulatory differences between l1 and l2 speakers of english. *Journal of Phonetics*, 70:86–116.

Wilcox, G., Gauthier, J., Hu, J., Qian, P., and Levy, R. (2020). On the predictive power of neural language models for human real-time comprehension behavior. *arXiv preprint arXiv:2006.01912*.

Wood, S. N. (2017). *Generalized Additive Models: An Introduction with R*. Chapman and Hall/CRC.

Wood, S. N. (2020). Inference and computation with generalized additive models and their extensions. *Test*, 29(2):307–339.

Wood, S. N., Pya, N., and Säfken, B. (2016). Smoothing parameter and model selection for general smooth models. *Journal of the American Statistical Association*, 111(516):1548–1563.
